# New Insights in Natural Bioactive Compounds for Periodontal Disease: Advanced Molecular Mechanisms and Therapeutic Potential

**DOI:** 10.3390/molecules30040807

**Published:** 2025-02-10

**Authors:** Nada Tawfig Hashim, Rasha Babiker, Nallan C. S. K. Chaitanya, Riham Mohammed, Sivan Padma Priya, Vivek Padmanabhan, Ayman Ahmed, Shahista Parveen Dasnadi, Md Sofiqul Islam, Bakri Gobara Gismalla, Muhammed Mustahsen Rahman

**Affiliations:** 1 Department of Periodontics, RAK College of Dental Sciences, RAK Medical & Health Sciences University, Ras-AlKhaimah 12973, United Arab Emirates; mustahsen@rakmhsu.ac.ae; 2 Department of Physiology, RAK College of Medical Sciences, RAK Medical & Health Science University, Ras-AlKhaimah 11127, United Arab Emirates; rashababiker@rakmhsu.ac.ae; 3 Department of Oral Medicine and Radiology, RAK College of Dental Sciences, RAK Medical & Health Sciences University, Ras-AlKhaimah 12973, United Arab Emirates; krishna.chytanya@rakmhsu.ac.ae; 4 Department Oral Surgery, RAK College of Dental Sciences, RAK Medical & Health Sciences University, Ras-AlKhaimah 12973, United Arab Emirates; riham.abdelraouf@rakmhsu.ac.ae; 5 Oral Pathology Department, RAK College of Dental Sciences, RAK Medical & Health Sciences University, Ras-AlKhaimah 12973, United Arab Emirates; sivan.padma@rakmhsu.ac.ae; 6 Department of Pediatric and Preventive Dentistry, RAK College of Dental Sciences, RAK Medical & Health Sciences University, Ras-AlKhaimah 12973, United Arab Emirates; vivek.padmanabhan@rakmhsu.ac.ae; 7 Department of Periodontology and Implantology, Nile University, Khartoum 1847, Sudan; ayman.ahmed@nileuniversity.edu.sd; 8 Department of Orthodontics, RAK College of Dental, RAK Medical & Health Sciences University, Ras-AlKhaimah 12973, United Arab Emirates; shahistha.parveen@rakmhsu.ac.ae; 9 Department of Operative Dentistry, RAK College of Dental Sciences, RAK Medical and Health Sciences University, Ras-AlKhaimah 12973, United Arab Emirates; sofiqul.islam@rakmhsu.ac.ae; 10 Department of Oral Rehabilitation, Faculty of Dentistry, University of Khartoum, Khartoum 11115, Sudan; bakri.gobara@uofk.edu

**Keywords:** periodontal disease, bioactive compounds, curcumin, resveratrol, EGCG (epigallocatechin gallate), baicalin, antimicrobial peptides, quorum sensing, immune modulation, efferocytosis, matrix metalloproteinases (MMPs)

## Abstract

Periodontal disease is a chronic inflammatory condition that destroys the tooth-supporting structures due to the host’s immune response to microbial biofilms. Traditional periodontal treatments, such as scaling and root planing, pharmacological interventions, and surgical procedures, have significant limitations, including difficulty accessing deep periodontal pockets, biofilm recolonization, and the development of antibiotic resistance. In light of these challenges, natural bioactive compounds derived from plants, herbs, and other natural sources offer a promising alternative due to their anti-inflammatory, antioxidant, antimicrobial, and tissue-regenerative properties. This review focuses on the molecular mechanisms through which bioactive compounds, such as curcumin, resveratrol, epigallocatechin gallate (EGCG), baicalin, carvacrol, berberine, essential oils, and Gum Arabic, exert therapeutic effects in periodontal disease. Bioactive compounds inhibit critical inflammatory pathways like NF-κB, JAK/STAT, and MAPK while activating protective pathways such as Nrf2/ARE, reducing cytokine production and oxidative stress. They also inhibit the activity of matrix metalloproteinases (MMPs), preventing tissue degradation and promoting healing. In addition, these compounds have demonstrated the potential to disrupt bacterial biofilms by interfering with quorum sensing, targeting bacterial cell membranes, and enhancing antibiotic efficacy.Bioactive compounds also modulate the immune system by shifting the balance from pro-inflammatory to anti-inflammatory responses and promoting efferocytosis, which helps resolve inflammation and supports tissue regeneration. However, despite the promising potential of these compounds, challenges related to their poor bioavailability, stability in the oral cavity, and the absence of large-scale clinical trials need to be addressed. Future strategies should prioritize the development of advanced delivery systems like nanoparticles and hydrogels to enhance bioavailability and sustain release, alongside long-term studies to assess the effects of these compounds in human populations. Furthermore, combining bioactive compounds with traditional treatments could provide synergistic benefits in managing periodontal disease. This review aims to explore the therapeutic potential of natural bioactive compounds in managing periodontal disease, emphasizing their molecular mechanisms of action and offering insights into their integration with conventional therapies for a more comprehensive approach to periodontal health.

## 1. Background

**Figure 1:**
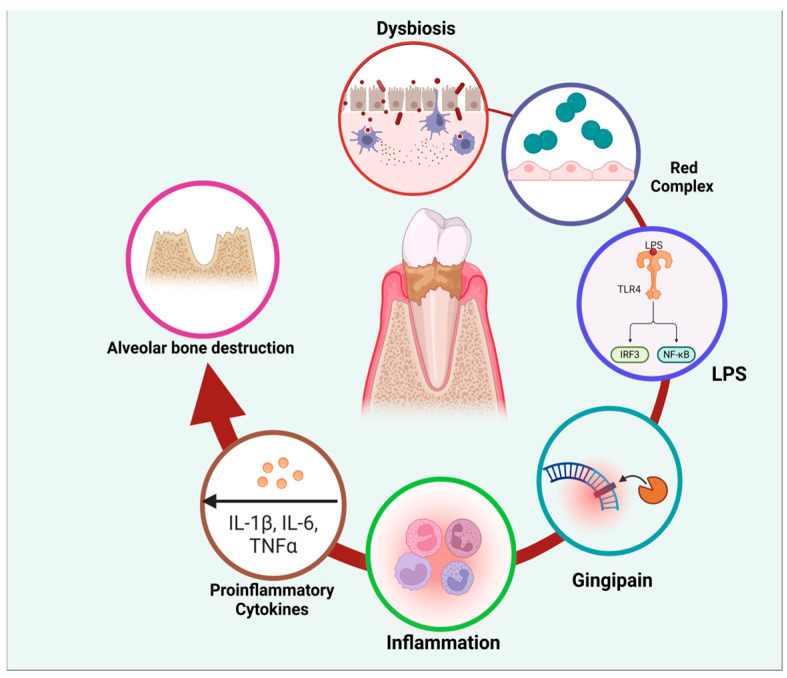
Illustrates the pathophysiological cycle of periodontitis, beginning with dysbiosis, where microbial imbalance initiates disease progression. The formation of biofilm and colonization by red-complex bacteria exacerbate inflammation through the release of lipopolysaccharides (LPS). This stimulates pro-inflammatory cytokines such as IL-1β, IL-6, and TNF-α, leading to gingival pain and progressive alveolar bone destruction. The inflammatory response further sustains dysbiosis, creating a vicious cycle that perpetuates tissue degradation and disease advancement. Created with BioRender.com.

Periodontal disease (PD) encompasses a range of inflammatory conditions that affect the supporting structures of the teeth, including the gingiva, periodontal ligament, cementum, and alveolar bone. PD is primarily driven by a dysbiotic microbial community within the subgingival biofilm [1]. While more than 500 bacterial species can be found in the oral cavity, a select group of pathogens known as the “red complex”, including *Porphyromonas gingivalis*, *Tannerella forsythia*, and *Treponema denticola*, are strongly associated with the initiation and progression of periodontitis [2]. These pathogens possess various virulence factors that contribute to their pathogenicity in periodontal disease [3]. Lipopolysaccharides (LPS) are key molecules that trigger strong inflammatory responses in the host, leading to tissue destruction [4]. Gingipains, which are proteolytic enzymes produced by P. gingivalis, play a crucial role in degrading host tissues and evading immune responses [5] (Figure 1).

**Figure 2:**
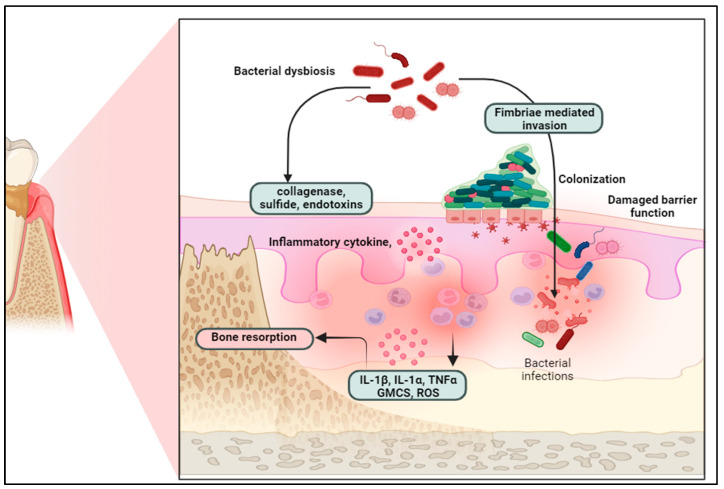
Illustration of bacterial dysbiosis leading to periodontal inflammation. Pathogenic bacteria invade via fimbriae-mediated adhesion, colonizing periodontal tissues and disrupting barrier function. This triggers an immune response, leading to the release of pro-inflammatory cytokines (IL-1β, IL-6, IL-1α, TNF-α) and ROS by pro-inflammatory cells, which include macrophages, neutrophils, and activated fibroblasts. Created with BioRender.com.

Additionally, fimbriae and adhesins facilitate the adhesion of these pathogens to host cells and other bacteria, promoting biofilm formation and enhancing their persistence within the periodontal environment [3]. Together, these factors contribute significantly to the progression of periodontal disease. The pathogenesis of periodontal disease (PD) is not solely due to bacterial infection but is predominantly influenced by the host’s immune-inflammatory response to these pathogens [2]. Periodontal disease begins with microbial colonization on the tooth surface, forming biofilms that mature and invade subgingival spaces [6]. This results in accumulating bacterial products, toxins, and metabolic byproducts, activating the host immune system [7]. Initially, gingivitis develops which is characterized by redness, swelling, and bleeding of the gingiva. If left untreated, this progresses to periodontitis, where chronic inflammation leads to irreversible destruction of the periodontal ligament and alveolar bone [8]. The pathophysiology of periodontal disease (PD) is driven by an overactive immune response to pathogenic bacteria, leading to the destruction of the tooth-supporting structures [2] (Figure 2).

Inflammatory cells form the first line of defense, initially involving neutrophils, followed by the recruitment of macrophages and lymphocytes. However, chronic exposure to bacterial antigens results in persistent activation of these immune cells, leading to the release of pro-inflammatory cytokines such as tumor necrosis factor-alpha (TNF-α), interleukin-1β (IL-1β), and IL-6 [9]. These cytokines not only sustain the inflammatory process but also activate osteoclasts through the receptor activator of the nuclear factor-kappa B ligand (RANKL) pathway, ultimately driving bone resorption [10,11]. In addition, matrix metalloproteinases (MMPs), including MMP-1, MMP-8, and MMP-9, are produced by neutrophils and fibroblasts in response to pro-inflammatory signals, degrading the extracellular matrix and contributing to tissue breakdown [12]. The inflammatory response also generates reactive oxygen species (ROS), which exacerbate tissue damage by promoting lipid peroxidation, DNA damage, and apoptosis in host cells. These combined processes lead to the progressive destruction of periodontal tissues [13].

Current treatments for periodontal disease primarily focus on reducing microbial load and controlling inflammation to halt tissue destruction. These therapies include mechanical debridement, pharmacological interventions, and surgical procedures, each with specific limitations [14].

Scaling and Root Planing (SRP)**,** a non-surgical mechanical debridement, aims to remove plaque and calculus from the tooth surface [15]. While effective in reducing bacterial load and improving clinical parameters such as probing depth and clinical attachment levels, SRP has several challenges. Deep periodontal pockets and furcation areas can be difficult to access, leading to incomplete biofilm removal [16]. Furthermore, SRP alone cannot fully eliminate bacterial pathogens, resulting in biofilm recolonization over time. The success of SRP also heavily depends on the clinician’s skill, which can lead to variability in outcomes [17].

Pharmacological interventions, such as systemic or locally delivered antibiotics (e.g., metronidazole or tetracyclines), are often used as adjuncts to mechanical therapy [18]. However, their use is limited by the development of antibiotic resistance, which occurs when antibiotics are overused or inappropriately prescribed, reducing their long-term effectiveness [19]. Additionally, antibiotics can disrupt the natural oral microbiome, leading to dysbiosis or the overgrowth of opportunistic pathogens. The effects of antibiotics are typically short-lived, as biofilms are resilient and can recolonize post-treatment [20]. For advanced periodontitis, surgical interventions, such as periodontal flap surgery, are employed to access and clean deep periodontal pockets and, in some cases, regenerate lost bone and tissue [21]. However, surgical procedures are invasive and often associated with postoperative discomfort and extended healing times, deterring patients from opting for them. Moreover, outcomes from surgery can vary, with bone or tissue regeneration being incomplete, particularly in cases with severe damage [22]. Surgical treatments also come with a high financial burden, making them inaccessible to some patients [23]. Despite these limitations, ongoing advancements in periodontal therapy aim to address these challenges by improving treatment precision, minimizing invasiveness, and enhancing long-term outcomes [24]. Most conventional therapies target the symptoms of PD (i.e., bacterial biofilms and inflammation) rather than addressing the underlying molecular mechanisms driving the disease. They do not fully restore immune homeostasis or resolve chronic inflammation, leaving patients susceptible to recurrent disease [25]. Furthermore, current treatments do not prevent the systemic effects of periodontal disease, such as its association with cardiovascular disease, diabetes, and adverse pregnancy outcomes, which suggests the need for therapeutic strategies with broader biological impacts [26,27]. These challenges necessitate the exploration of innovative therapeutic avenues that target the underlying molecular mechanisms of periodontal disease. Natural bioactive compounds derived from plants, herbs, and other natural sources offer a compelling alternative, demonstrating anti-inflammatory, antioxidant, antimicrobial, and tissue-regenerative properties. By modulating key signaling pathways such as NF-κB and Nrf2/ARE, bioactive compounds provide a holistic approach to managing periodontal inflammation and promoting tissue repair [28]. This review discusses the therapeutic potential of natural bioactive compounds, highlighting their molecular mechanisms of action and proposing their integration with traditional periodontal therapies for enhanced clinical outcomes.

## 2. Natural Bioactive Compounds as Emerging Alternatives

Bioactive compounds derived from plants, herbs, and other natural sources display a variety of beneficial biological activities, including anti-inflammatory, antioxidant, antimicrobial, and tissue-regenerative properties [29]. These compounds—such as curcumin, resveratrol, quercetin, catechins, baicalin, carvacrol, β-caryophyllene, berberine, essential oils, antimicrobial peptides (AMPs), and Gum Arabic—offer significant advantages over conventional treatments [30,31]. Targeting microbial pathogens and modulating the host immune response effectively intervene in key signaling pathways, enzymes, and cellular processes associated with periodontal disease progression, making them promising agents in enhancing periodontal health [32].

The therapeutic properties of bioactive compounds in managing periodontal disease stem from a wide range of natural sources, with each source contributing uniquely to their beneficial effects [28]. These diverse origins allow the compounds to offer specific and targeted actions in periodontal therapy [28]. Curcumin is derived from the turmeric plant (*Curcuma longa*), while resveratrol is found in grapes, berries, and red wine [33]. Quercetin, a potent flavonoid, is widely present in fruits and vegetables like apples, onions, and citrus fruits [34]. Catechins, particularly EGCG, are primarily sourced from green tea (*Camellia sinensis*) [35]. Baicalin is extracted from the roots of *Scutellaria baicalensis* (*Chinese skullcap*), and carvacrol, a monoterpenoid phenol, is found in oregano (*Origanum vulgare*) and thyme (*Thymus vulgaris*) [36,37]. β-Caryophyllene is a sesquiterpene derived from essential oils of black pepper (*Piper nigrum*) and clove (*Syzygium aromaticum*), while berberine, a potent alkaloid, is sourced from plants such as barberry (*Berberis vulgaris*) and goldthread (*Coptis chinensis*) [38]. Thymol and eucalyptol, commonly found in essential oils, are extracted from thyme and eucalyptus (*Eucalyptus globulus*), respectively [39]. Antimicrobial peptides (AMPs) occur naturally in the human body or are synthesized in laboratories, and Gum Arabic, a natural gum, is derived from the sap of the Acacia tree (*Acacia senegal* or *Acacia seyal*) [31,40]. These natural compounds provide a wide array of therapeutic benefits for the treatment and management of periodontal disease [28,31] (Table 1).

## 3. Molecular Mechanisms of Action of Natural Bioactive Compounds

### 3.1. Modulation of Inflammatory Pathways

**Inhibition of NF-κB signaling**: Many bioactive compounds suppress NF-κB, a critical transcription factor that regulates pro-inflammatory cytokines like TNF-α, IL-6, and IL-1β. Inhibition of the NF-κB (Nuclear factor kappa-light-chain-enhancer of activated B cells) signaling pathway is a crucial mechanism through which many bioactive compounds exert their anti-inflammatory effects [41]. NF-κB is a transcription factor that plays a central role in regulating the immune response to infection and inflammation. Under normal conditions, NF-κB is sequestered in the cytoplasm by its inhibitor, IκB (Inhibitor of κB), which prevents its translocation to the nucleus [42]. However, in response to various stimuli, such as bacterial antigens, cytokines, oxidative stress, and inflammatory mediators, IκB is phosphorylated and subsequently degraded by the proteasome, allowing NF-κB to enter the nucleus and activate the transcription of pro-inflammatory genes [41]. Once inside the nucleus, NF-κB binds to specific DNA sequences in the promoter regions of genes that encode for pro-inflammatory cytokines, including tumor necrosis factor-alpha (TNF-α), interleukin-6 (IL-6), and interleukin-1β (IL-1β) [43]. These cytokines are major contributors to the inflammatory response, leading to the recruitment of immune cells, increased production of ROS, and activation of additional pro-inflammatory pathways, which collectively result in tissue destruction in conditions such as periodontal disease [44]. NF-κB also regulates the expression of enzymes such as cyclooxygenase-2 (COX-2) and inducible nitric oxide synthase (iNOS), both of which exacerbate inflammation by promoting the production of inflammatory mediators like prostaglandins and nitric oxide [45].

**Figure 3:**
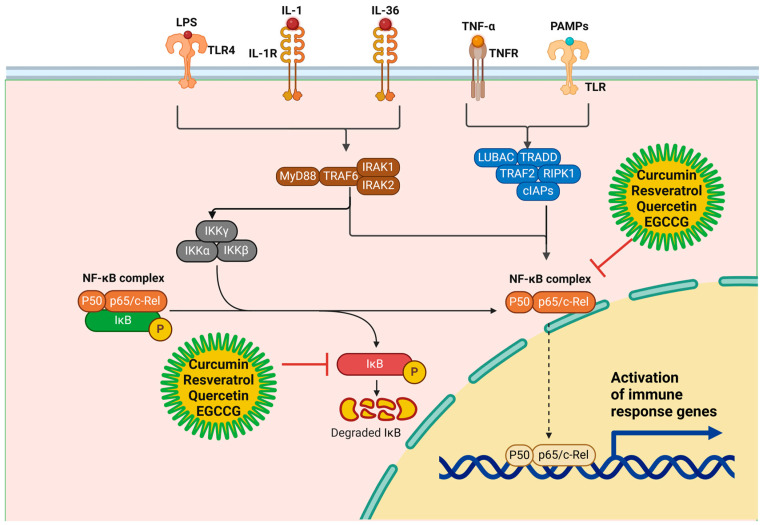
Illustrates the NF-κB signaling pathway, highlighting the role of pro-inflammatory stimuli such as LPS, IL-1, IL-36, TNF-α, and PAMPs, which activate receptors (TLR4, IL-1R, TNFR) to initiate intracellular signaling cascades. Activation of adaptor proteins (MyD88, TRAF6, IRAK1/2) leads to phosphorylation and degradation of IκB, freeing the NF-κB complex (p50/p65) to translocate into the nucleus and activate immune response genes. Curcumin acts as an inhibitor at multiple points, preventing IκB degradation and blocking NF-κB nuclear translocation, thereby reducing inflammation and modulating immune responses. Created with BioRender.com.

Bioactive compounds, including curcumin, resveratrol, quercetin, and EGCG, have been shown to suppress NF-κB activity through various mechanisms [46,47,48,49]. For instance, curcumin inhibits the phosphorylation and degradation of IκB, thereby preventing NF-κB from translocating to the nucleus. This effectively blocks the transcription of pro-inflammatory cytokines and reduces the inflammatory response [50]. Resveratrol similarly inhibits IκB kinase (IKK), which is responsible for the phosphorylation of IκB, thereby maintaining NF-κB in its inactive cytoplasmic form. Additionally, these compounds can inhibit upstream signaling molecules such as TNF receptor-associated factor (TRAF) and mitogen-activated protein kinases (MAPKs), which are involved in the activation of NF-κB in response to external stimuli [43]. By inhibiting NF-κB signaling, these bioactive compounds help to reduce the expression of pro-inflammatory cytokines, decrease immune cell recruitment, and limit the production of ROS and other inflammatory mediators [51,52]. The ability of these compounds to modulate NF-κB signaling makes them promising therapeutic agents for controlling chronic inflammation and preventing the progression of inflammatory diseases (Figure 3).

**Activation of Nrf2/ARE pathways**: The Nrf2 (Nuclear factor erythroid 2-related factor 2) pathway is a pivotal component of the body’s antioxidant defense system, acting as a transcription factor that regulates the expression of numerous genes responsible for protecting cells from oxidative stress [53]. Upon activation, Nrf2 enhances the production of antioxidant enzymes, detoxification proteins, and other cytoprotective molecules that collectively neutralize reactive oxygen species (ROS) and maintain cellular redox balance [53]. Among the key antioxidant enzymes regulated by Nrf2 are superoxide dismutase (SOD), which converts superoxide anions into less reactive hydrogen peroxide; catalase, which breaks down hydrogen peroxide into water, oxygen, and glutathione peroxidase (GPx), which neutralizes hydrogen peroxide and lipid peroxides, preventing oxidative damage to cellular components such as lipids, proteins, and DNA [54,55]. Additionally, Nrf2 plays a crucial role in detoxifying reactive molecules by regulating phase II detoxifying enzymes, such as glutathione S-transferase (GST), which conjugates glutathione to reactive intermediates, and NAD(P)H: quinone oxidoreductase 1 (NQO1), which reduces quinones to less reactive hydroquinones [55]. Nrf2 also helps maintain cellular redox balance by promoting the synthesis of glutathione (GSH), a key antioxidant molecule, through the upregulation of enzymes like glutamate-cysteine ligase (GCL), which is essential for glutathione production [55]. Furthermore, Nrf2 protects cells against inflammation and apoptosis by activating antioxidant and detoxification pathways, thereby reducing oxidative stress that can lead to chronic inflammation. This is particularly relevant in the context of chronic diseases such as cardiovascular disease, neurodegenerative disorders, and inflammatory conditions like periodontal disease [56]. Under normal conditions, Nrf2 is sequestered in the cytoplasm by its inhibitor, Keap1 (Kelch-like ECH-associated protein 1), which promotes Nrf2’s ubiquitination and degradation. However, in response to oxidative stress or exposure to electrophilic compounds, the Keap1-Nrf2 complex is disrupted, allowing Nrf2 to translocate to the nucleus [57]. Once in the nucleus, Nrf2 binds to the Antioxidant Response Element (ARE) in the promoter regions of target genes, initiating the transcription of genes involved in antioxidant defense and cytoprotection [55]. In periodontal disease, oxidative stress plays a significant role in tissue destruction and disease progression [58]. Nrf2 activation can mitigate these effects by boosting the expression of antioxidant enzymes, thereby reducing the harmful impacts of ROS in periodontal tissues [59].

**Figure 4:**
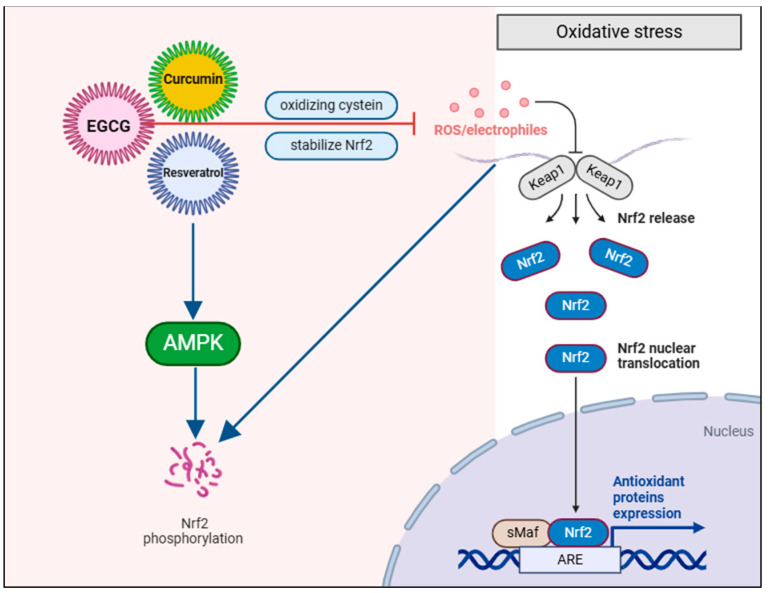
Illustrates the Nrf2 (nuclear factor erythroid 2-related factor 2) signaling pathway in response to oxidative stress. Under normal conditions, Nrf2 is bound to Keap1 in the cytoplasm, leading to its degradation. Upon oxidative stress or exposure to ROS/electrophiles, Nrf2 dissociates from Keap1 and translocates into the nucleus, where it binds to antioxidant response elements (ARE) to promote the expression of antioxidant proteins. Phytochemicals such as curcumin, EGCG, and resveratrol enhance Nrf2 activation by stabilizing Nrf2, oxidizing cysteine residues, or activating AMPK, which phosphorylates Nrf2. This pathway plays a crucial role in cellular defense mechanisms against oxidative damage. Created with BioRender.com.

Bioactive compounds like curcumin, resveratrol, and EGCG have been shown to activate the Nrf2 pathway, enhancing antioxidant defenses and protecting periodontal cells from ROS-induced damage [60]. Curcumin induces electrophilic stress that disrupts the Keap1-Nrf2 complex by oxidizing cysteine residues on Keap1, allowing Nrf2 to translocate into the nucleus and promote the expression of antioxidant enzymes like superoxide dismutase (SOD) and catalase [60]. Similarly, resveratrol also disrupts the Keap1-Nrf2 interaction but additionally activates the AMPK pathway, which enhances Nrf2 phosphorylation, further promoting its nuclear translocation and the expression of enzymes like glutathione-S-transferase (GST) and heme oxygenase-1 (HO-1) [61]. EGCG, the main catechin in green tea, shares this mechanism of Keap1 disruption but also activates protein kinase pathways, such as ERK and PI3K/Akt, which further stabilize Nrf2 and enhance its retention in the nucleus, promoting the production of antioxidant enzymes [62]. While they all share the goal of activating Nrf2 and boosting antioxidant defenses, the pathways they influence highlight subtle differences in their mechanisms (Figure 4).

In addition to curcumin, resveratrol, and EGCG, quercetin has also been shown to activate the Nrf2 pathway, contributing to its antioxidant and cytoprotective effects. Quercetin disrupts the Keap1-Nrf2 complex, leading to the nuclear translocation of Nrf2 and the subsequent upregulation of antioxidant enzymes such as superoxide dismutase (SOD), catalase, glutathione-S-transferase (GST), and heme oxygenase-1 (HO-1) [63]. Furthermore, quercetin has been found to modulate additional pathways such as PI3K/Akt and MAPK, which further stabilize Nrf2 and enhance its transcriptional activity. By reducing oxidative stress and inflammation, quercetin plays a crucial role in protecting osteoblasts from oxidative damage, inhibiting osteoclastogenesis, and maintaining bone homeostasis. Given its antioxidant potential, quercetin may serve as a valuable adjunct in the management of periodontitis by mitigating oxidative stress-induced alveolar bone loss and promoting periodontal tissue regeneration [64,65].

**Modulation of JAK/STAT and MAPK pathways**: Key signaling cascades involved in inflammation and immune responses are targeted by bioactive compounds, leading to reduced tissue destruction. The JAK/STAT pathway is activated in response to cytokines and growth factors that bind to their respective receptors on the cell surface [66]. Upon cytokine binding, receptor-associated JAK proteins are activated through phosphorylation, which in turn phosphorylates STAT proteins [66]. Phosphorylated STAT proteins dimerize and translocate to the nucleus, where they bind to DNA and initiate the transcription of target genes involved in inflammation, immune regulation, and cellular growth [67]. Bioactive compounds have been shown to inhibit the JAK/STAT pathway by interfering with various components of this signaling cascade [68]. Curcumin exerts its anti-inflammatory effects by inhibiting the JAK/STAT (Janus kinase/signal transducer and activator of transcription) signaling pathway, which is essential in mediating immune responses and the production of pro-inflammatory cytokines. Specifically, curcumin blocks the phosphorylation of JAKs (Janus kinases) and STAT3 (signal transducer and activator of transcription 3) [69]. This inhibition prevents the phosphorylation and dimerization of STAT3, which is necessary for its translocation to the nucleus, where it activates the transcription of pro-inflammatory genes. As a result, the production of pro-inflammatory cytokines like interleukin-6 (IL-6) and interferon-γ (IFN-γ) is significantly reduced [69]. These cytokines are key players in promoting inflammation and recruiting immune cells to inflamed tissues, so curcumin’s inhibition of this pathway effectively suppresses the immune response and reduces inflammation. Additionally, by inhibiting the JAK/STAT pathway, curcumin modulates other downstream inflammatory mediators, contributing to its broad-spectrum anti-inflammatory action [69].

Resveratrol, similarly, affects the JAK/STAT pathway but specifically targets JAK2 and STAT3. Resveratrol inhibits the phosphorylation and activation of JAK2, which in turn prevents STAT3 activation [70]. By disrupting this pathway, resveratrol reduces the expression of pro-inflammatory cytokines and inhibits immune cell recruitment to sites of inflammation. This reduction in immune cell infiltration is critical for controlling tissue damage caused by excessive inflammation. Moreover, resveratrol’s inhibition of STAT3 not only reduces the expression of pro-inflammatory mediators like IL-6 but also influences other signaling pathways, such as the NF-κB pathway, which further amplifies its anti-inflammatory effects. Resveratrol has also been shown to activate AMPK (AMP-activated protein kinase)**,** which enhances its anti-inflammatory properties by promoting cellular energy balance and reducing the inflammatory response [70].

**Figure 5:**
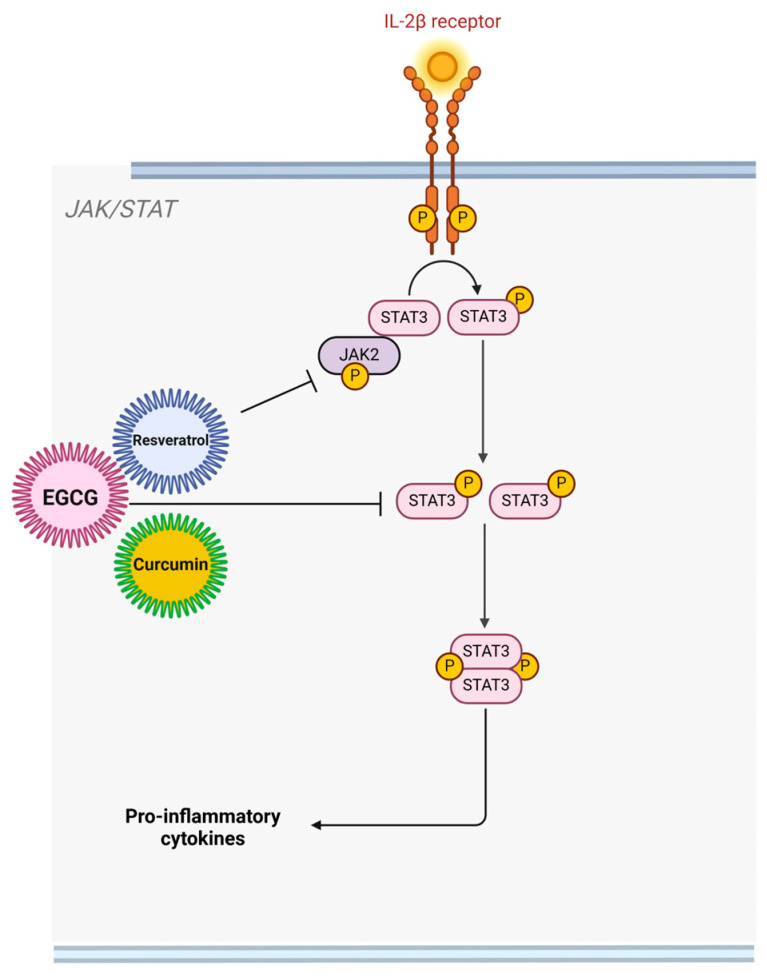
Depicts the JAK/STAT signaling pathway activated by the IL-2 receptor, leading to the phosphorylation and dimerization of STAT3 proteins. Upon receptor activation, JAK2 phosphorylates STAT3, which subsequently dimerizes and translocates into the nucleus to promote the expression of pro-inflammatory cytokines. The pathway highlights the inhibitory effects of curcumin, resveratrol, and EGCG, which interfere with STAT3 phosphorylation and nuclear translocation, thereby reducing inflammation. Created with BioRender.com.

Both curcumin and resveratrol work to downregulate the JAK/STAT signaling pathway, but their specific targets within the pathway differ slightly. While both compounds inhibit STAT3 activation, curcumin broadly affects JAK kinases, whereas resveratrol specifically inhibits JAK2 [69,70]. This nuanced difference contributes to their varied impacts on inflammatory mediators, making both compounds potent inhibitors of inflammation with potential synergistic effects when used together (Figure 5).

**Figure 6:**
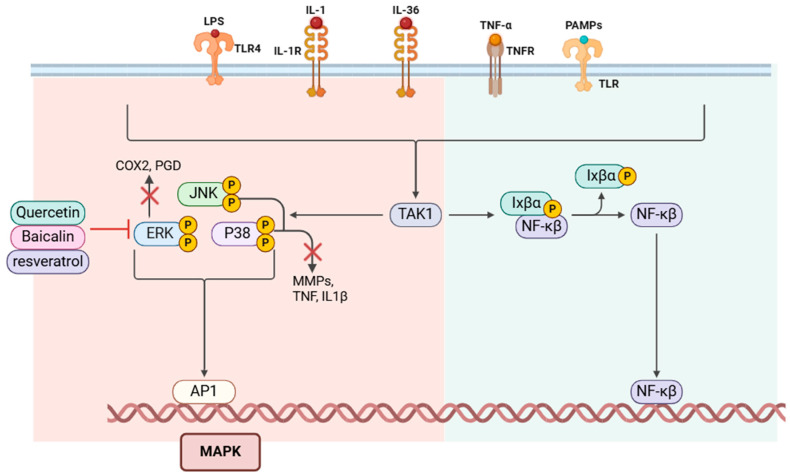
Illustrates the MAPK and NF-κB signaling pathways activated by pro-inflammatory stimuli such as LPS, IL-1, IL-26, TNF-α, and PAMPs. Upon activation, the MAPK pathway phosphorylates ERK, JNK, and P38, leading to the transcription of pro-inflammatory mediators (COX2, PGD, MMPs, TNF, and IL-1β) through AP1. Concurrently, the NF-κB pathway involves phosphorylation and degradation of IκBα, enabling NF-κB to translocate into the nucleus and drive inflammatory gene expression. The figure highlights the inhibitory effects of quercetin, baicalin, and resveratrol, which block phosphorylation in the MAPK pathway, reducing inflammation and cytokine production. Created with BioRender.com.

- The MAPK pathway is another key signaling cascade that is activated in response to extracellular stimuli such as stress, cytokines, and microbial components. It regulates a variety of cellular processes, including inflammation, proliferation, differentiation, and apoptosis [71]. The MAPK pathway involves a series of phosphorylation events mediated by three major groups of MAPKs: ERK (extracellular signal-regulated kinase), JNK (c-Jun N-terminal kinase), and p38 MAPK. These kinases are activated in response to pro-inflammatory signals and subsequently regulate the transcription of genes involved in inflammation and tissue remodeling [71]. Quercetin, baicalin, and resveratrol are known to modulate the MAPK pathway by inhibiting the phosphorylation and activation of ERK, JNK, and p38 MAPK [72]. Quercetin, for instance, reduces the activation of p38 MAPK, which in turn decreases the production of inflammatory cytokines like TNF-α and IL-1β [63]. Baicalin inhibits the JNK and p38 pathways, thereby reducing the production of matrix metalloproteinases (MMPs) that contribute to tissue destruction in periodontal disease [73]. Resveratrol has been shown to inhibit the phosphorylation of ERK, reducing the expression of COX-2 and prostaglandins, which are key mediators of inflammation [74]. By modulating the JAK/STAT and MAPK pathways, bioactive compounds help to control the excessive inflammatory response and prevent tissue destruction in conditions such as periodontal disease. These signaling cascades play pivotal roles in driving chronic inflammation, and their inhibition by natural compounds provides a promising approach to managing inflammatory diseases and promoting tissue repair [28] (Figure 6).

### 3.2. Influence on Matrix Metalloproteinases (MMPs)

Matrix metalloproteinases (MMPs) are a family of proteolytic enzymes that play a critical role in the degradation of extracellular matrix (ECM) components, which is a hallmark of tissue destruction in periodontal disease [75]. Among the various MMPs, MMP-2 (gelatinase A) and MMP-9 (gelatinase B) are particularly involved in the breakdown of collagen, the major structural protein in periodontal tissues [76]. Excessive MMP activity, driven by chronic inflammation, leads to the degradation of collagen and other ECM components, contributing to the progression of periodontitis and the subsequent loss of tooth-supporting structures [75].

Bioactive compounds such as curcumin and resveratrol have demonstrated significant potential in downregulating the expression and activity of MMPs, particularly MMP-2 and MMP-9 [77,78]. Curcumin inhibits the AP-1 transcription factor, which is involved in the regulation of MMP expression, particularly MMP-2 and MMP-9. Curcumin also inhibits the activation of mitogen-activated protein kinases (MAPKs), which play a crucial role in the signaling pathways that lead to inflammation and MMP production. By suppressing these pathways, curcumin effectively reduces the production of pro-inflammatory cytokines and MMPs, limiting tissue degradation. Moreover, curcumin’s potent antioxidant properties further protect tissues from oxidative stress, which can exacerbate MMP activity [79]. Resveratrol suppresses NF-κB activation, which reduces the transcription of pro-inflammatory cytokines such as TNF-α and IL-6, thereby inhibiting the downstream expression of MMPs. Additionally, resveratrol enhances the activity of tissue inhibitors of metalloproteinases (TIMPs), which naturally regulate MMP activity, further suppressing excessive tissue degradation. This dual action—reducing MMP expression and boosting TIMP activity—makes resveratrol particularly effective in controlling periodontal inflammation and preventing tissue breakdown [78].

Overall, by downregulating the expression of MMP-2 and MMP-9, natural compounds like resveratrol and curcumin help maintain the balance between tissue breakdown and repair in periodontal disease, offering a promising approach to preserving periodontal tissue integrity and preventing further progression of the disease [78,79].

### 3.3. Epigenetic Modifications

Natural bioactive compounds have been shown to modulate gene expression through epigenetic mechanisms, such as DNA methylation and histone acetylation, which are key regulators of chromatin structure and gene transcription [80]. These epigenetic modifications can influence a wide range of biological processes, including inflammation, cell proliferation, and tissue repair [81]. One of the most well-studied compounds in this context is curcumin, which exerts its effects by altering the activity of histone deacetylases (HDACs) [82]. Histone deacetylases (HDACs) play a critical role in regulating gene expression by modifying the acetylation status of histones, the proteins around which DNA is wound [83]. When HDACs remove acetyl groups from histones, it results in a more condensed chromatin structure, making it difficult for transcription factors to access the DNA and initiate gene expression [84]. This deacetylated state is often associated with the repression of genes, including those involved in anti-inflammatory and regenerative processes [85].

Curcumin, by inhibiting HDAC activity, promotes the acetylation of histones, particularly at lysine residues [86]. This acetylation weakens the interaction between histones and DNA, resulting in a more relaxed or “open” chromatin structure. This open chromatin configuration makes it easier for transcription factors and the transcriptional machinery to access promoter regions of genes that are involved in anti-inflammatory responses and tissue regeneration [87,88]. For instance, genes encoding for anti-inflammatory cytokines (e.g., IL-10) or tissue-protective enzymes can be more actively transcribed [89]. Additionally, curcumin’s ability to inhibit HDACs enhances the expression of tumor suppressor genes and other protective factors, contributing to its overall anti-inflammatory and tissue-regenerative effects [90]. This epigenetic modulation of gene expression allows curcumin to exert long-lasting beneficial effects on cellular function, particularly in inflammatory conditions like periodontitis or other chronic diseases [82].

Moreover, curcumin has also been shown to influence DNA methylation by modulating the activity of DNA methyltransferases (DNMTs)**,** enzymes responsible for adding methyl groups to DNA, which typically silences gene expression [91]. Through these dual mechanisms—HDAC inhibition and DNMT modulation—curcumin helps regulate gene expression in a way that reduces inflammation and promotes periodontal tissue integrity [91].

### 3.4. Regulation of Apoptosis and Cell Proliferation

Bioactive compounds have been shown to modulate apoptotic pathways in both host cells and pathogenic bacteria, playing a crucial role in managing cellular health and maintaining tissue integrity, especially in the context of chronic inflammatory diseases like periodontal disease (28). Apoptosis, or programmed cell death, is a tightly regulated process that helps eliminate damaged or infected cells, thereby preventing further tissue damage and promoting healing [92]. For instance, quercetin has been shown to induce apoptosis in gingival fibroblasts that are exposed to oxidative stress [93]. Quercetin achieves this by modulating the balance between pro-apoptotic and anti-apoptotic proteins, particularly by influencing the Bcl-2/Bax ratio. The Bcl-2 family of proteins plays a key role in regulating apoptosis: Bcl-2 is an anti-apoptotic protein that promotes cell survival, while Bax is a pro-apoptotic protein that promotes cell death [94]. Under conditions of oxidative stress, quercetin downregulates Bax expression while upregulating Bcl-2, thereby promoting the survival of healthy cells and inducing apoptosis in damaged cells [94]. This selective regulation of apoptosis ensures that unhealthy or damaged cells are removed, while healthy cells are preserved, contributing to tissue homeostasis and repair [94].

## 4. Microbial Interaction and Biofilm Disruption of Natural Bioactive Compounds

Natural bioactive compounds have demonstrated significant potential in targeting bacterial biofilms by interfering with bacteria’s communication systems to coordinate biofilm formation and virulence. These compounds disrupt key molecular pathways that bacteria rely on, effectively reducing biofilm stability and bacterial pathogenicity [95].

### 4.1. Inhibition of Quorum Sensing in Bacteria

Quorum sensing (QS) is a crucial bacterial communication mechanism that regulates gene expression based on cell population density, enabling bacteria to coordinate activities like biofilm formation and virulence factor production [96]. In periodontal pathogens, quorum sensing plays an essential role in biofilm architecture, the production of extracellular polymeric substances (EPS), and the expression of virulence factors such as proteases and toxins (94) [96]. Interference with this system can disrupt bacterial coordination, making it an attractive target for reducing biofilm stability and pathogenicity in periodontal diseases [97].

Carvacrol exerts its quorum-sensing inhibitory effects on *Porphyromonas gingivalis* by specifically interfering with the production and sensing of autoinducer-2 (AI-2), a universal signaling molecule used in bacterial communication [98]. AI-2 is synthesized by the enzyme LuxS, which is part of the methyl cycle in bacterial cells. AI-2 signaling regulates the expression of genes involved in biofilm formation, virulence factor production, and extracellular polymeric substance (EPS) synthesis, all of which contribute to biofilm stability and pathogenicity [99].

At the molecular level, carvacrol disrupts AI-2-mediated quorum sensing by inhibiting the activity of LuxS, preventing the accumulation of AI-2 molecules in the extracellular environment [100]. Without sufficient AI-2, the bacteria cannot effectively communicate to coordinate biofilm development and virulence expression. This disruption in signaling leads to the downregulation of key genes responsible for EPS production, specifically those involved in synthesizing exopolysaccharides and extracellular proteins, essential biofilm matrix components [101]. By weakening the biofilm’s structural integrity, carvacrol renders *P. gingivalis* more susceptible to mechanical disruption (such as scaling and root planing) [102]. It enhances the efficacy of antimicrobial agents that would otherwise struggle to penetrate the protective biofilm. Thus, carvacrol’s ability to inhibit quorum sensing through AI-2 interference is a promising mechanism for reducing biofilm formation and controlling periodontal infections at the molecular level [102].

Resveratrol also disrupts quorum sensing in *Aggregatibacter actinomycetemcomitans*, a key periodontal pathogen, by targeting the LuxS/AI-2 system [103]. This disruption inhibits biofilm formation and reduces the bacteria’s ability to adhere to tooth surfaces [104]. While resveratrol shares a similar mechanism with carvacrol, it specifically acts on *A. actinomycetemcomitans*, illustrating the unique targeting capabilities of different bioactive compounds. Both resveratrol and carvacrol highlight the potential of natural compounds to interfere with bacterial communication systems, effectively reducing biofilm formation and virulence, which are central to the progression of periodontal disease [105].

### 4.2. Direct Antimicrobial Activity

Beyond quorum sensing inhibition, many bioactive compounds possess direct bactericidal effects on periodontal pathogens by targeting critical microbial processes, including cell membrane integrity, DNA synthesis, and essential enzymatic pathways [28].

Berberine, an alkaloid derived from *Berberis vulgaris*, has shown potent antimicrobial activity against key periodontal pathogens such as *Porphyromonas gingivalis, Fusobacterium nucleatum*, and *Tannerella forsythia* [106]. Berberine exerts its antimicrobial effects primarily by disrupting bacterial cell membranes, leading to increased membrane permeability and subsequent bacterial lysis [107]. In addition to its membrane-targeting actions, berberine inhibits crucial enzymes involved in bacterial energy metabolism, such as ATPase [105]. This inhibition results in a significant collapse in cellular ATP levels, depriving the bacteria of the energy needed for survival and leading to rapid bacterial death [108]. Moreover, recent advancements in the formulation of berberine-loaded nanoparticles have enhanced its bioavailability and allowed for targeted delivery to periodontal sites, further amplifying its antimicrobial efficacy against biofilm-embedded pathogens [109].

Catechins, particularly epigallocatechin gallate (EGCG) from green tea, possess a complex antimicrobial action against periodontal pathogens [110]. EGCG disrupts the bacterial lipid bilayers by embedding itself into the membrane. The exact mechanism by which epigallocatechin gallate (EGCG) interacts with bacterial lipid bilayers is attributed to its specific molecular structure. EGCG contains multiple hydroxyl (-OH) groups on its phenolic rings, which are hydrophilic, and it also has planar aromatic rings that can interact with hydrophobic regions [111]. The aromatic rings interact with the lipid bilayer by embedding themselves within the fatty acyl chains of the membrane, disrupting hydrophobic interactions that hold the membrane together [112].

Meanwhile, the hydroxyl groups of EGCG can form hydrogen bonds with polar head groups of the lipids on the membrane surface, further destabilizing the lipid arrangement. This dual interaction—hydrophobic insertion and polar interaction—causes the membrane to lose structural integrity, leading to increased permeability and ultimately bacterial cell lysis [113]. The lipid bilayer disruption is a key part of EGCG’s broad-spectrum antimicrobial action [113]. Beyond membrane disruption, EGCG specifically targets virulence factors in *Porphyromonas gingivalis*, such as gingipains, which are cysteine proteases critical for tissue invasion and immune evasion. By binding to these proteases, EGCG inhibits their enzymatic activity, thereby preventing the degradation of host tissues and protecting against immune suppression [114,115]. Additionally, EGCG acts as a metal ion chelator, particularly binding to essential cofactors like iron and zinc that bacteria require for activating various enzymes. By depriving the bacteria of these critical ions, EGCG inhibits key bacterial enzymatic processes, such as DNA replication, protein synthesis, and oxidative stress defense [116]. This multifaceted inhibition of bacterial growth and virulence positions EGCG as a potent therapeutic agent in periodontal disease management [116].

### 4.3. Synergistic Effects of Antibiotics

The rising concern of antibiotic resistance in the treatment of periodontal disease has prompted investigations into the synergistic effects of natural bioactive compounds with conventional antibiotics. By enhancing antibiotic efficacy, these compounds can lower the required dosage and minimize the potential for resistance development [117,118,119].

Curcumin has demonstrated synergy with antibiotics such as metronidazole and amoxicillin against *P. gingivalis* and *T. denticola*. By disrupting bacterial cell membranes and inhibiting biofilm formation, curcumin enhances the penetration of antibiotics into biofilm matrices. Recent research has shown that curcumin downregulates efflux pump genes in bacteria, a major mechanism by which pathogens develop resistance to antibiotics. This action allows antibiotics to accumulate in bacterial cells at higher concentrations, improving their bactericidal effects [120,121,122].

A combination of resveratrol and tetracycline has been shown to have synergistic effects against A. actinomycetemcomitans and *P. gingivalis* [105]. Resveratrol enhances the antibacterial activity of tetracycline by disrupting bacterial membranes and inhibiting the expression of resistance genes. This combination has demonstrated enhanced biofilm eradication in in vitro models, suggesting its potential as a combination therapy for recalcitrant periodontal infections [123].

### 4.4. Disruption of Biofilm Structure and Matrix

The extracellular polymeric substance (EPS) matrix is a vital element of bacterial biofilms, offering physical protection to the embedded pathogens and limiting the effectiveness of antimicrobial agents [124]. Carvacrol and thymol disrupt the biofilm matrix by degrading key components such as polysaccharides and proteins [125,126].

One of the key mechanisms involves the integration of thymol and carvacrol into the lipid bilayer of bacterial membranes [126]. These compounds possess hydroxyl groups that interact with the hydrophobic core of the phospholipid bilayer, disrupting the packing of lipid molecules. This action increases membrane fluidity and permeability, causing essential ions like potassium (K^+^) and hydrogen (H^+^) to leak out, while intracellular contents are also released. This loss of membrane integrity not only impairs bacterial cell function but also weakens the biofilm structure, making it more vulnerable to treatment [127].

In addition to membrane disruption, thymol and carvacrol inhibit the synthesis of polysaccharides, a key component of the EPS matrix. These compounds interfere with enzymes such as glucosyltransferases, which are crucial for the production of glucans that provide structural stability to the biofilm. The resulting reduction in glucan production compromises bacterial adhesion and biofilm cohesion, rendering the biofilm more susceptible to mechanical disruption and antimicrobial agents [128].

Moreover, carvacrol has been shown to specifically affect extracellular DNA (eDNA), a critical structural component of the EPS matrix that helps bind bacterial cells together. Carvacrol disrupts the interactions of eDNA, weakening the biofilm and making it easier for antimicrobial agents and host immune responses to penetrate and attack the bacteria [129,130].

By targeting these key components of the biofilm matrix, thymol and carvacrol effectively destabilize biofilm integrity. This disruption enhances the penetration of antimicrobial treatments and mechanical removal methods like scaling and root planing, ultimately improving the overall efficacy of periodontal therapy [131].

Baicalin, a flavonoid derived from *Scutellaria baicalensis* (also known as Chinese skullcap), specifically inhibits the synthesis of polysaccharides that are essential for biofilm formation and stability. Its action focuses primarily on the disruption of the enzymes responsible for the production of these polysaccharides, particularly glucans and other carbohydrate polymers that form the structural backbone of the extracellular polymeric substance (EPS) in bacterial biofilms [36].

At the molecular level, baicalin interferes with the activity of glucosyltransferases (Gtfs), which are enzymes critical for the biosynthesis of glucans [132,133]. These glucans serve as the scaffold for biofilm formation, allowing bacterial cells to adhere to surfaces and each other. By inhibiting Gtf activity, baicalin reduces the production of these essential polysaccharides, weakening the biofilm matrix. Without sufficient glucans, the bacterial cells lose their ability to maintain strong adhesion and cohesion within the biofilm [134].

Furthermore, baicalin disrupts the assembly of other components within the biofilm matrix, including extracellular polysaccharides like levan and fructans, which contribute to biofilm density and resilience. By interfering with the metabolic pathways involved in polysaccharide synthesis, baicalin hampers the accumulation of these carbohydrates, leading to a destabilized biofilm structure. This impairment makes bacterial biofilms more susceptible to both mechanical removal and antimicrobial treatments [135].

Another key aspect of baicalin’s action is its ability to reduce the expression of genes responsible for polysaccharide production. Baicalin modulates bacterial gene expression by affecting quorum sensing mechanisms, particularly by downregulating the genes involved in polysaccharide biosynthesis and biofilm maturation. This inhibition of signaling pathways further compromises the bacteria’s ability to produce and maintain a robust biofilm matrix [136].

Carvacrol, thymol, and baicalin disrupt the EPS matrix through slightly different mechanisms, yet they achieve the common outcome of making bacterial biofilms more vulnerable to external treatments [137].

## 5. Immunomodulation and Host-Microbial Homeostasis

Immunomodulation is a critical aspect of the body’s response to periodontal pathogens, which orchestrates the balance between protective immune responses and destructive inflammation [138]. Periodontal disease progression is heavily influenced by the dysregulation of immune responses, leading to chronic inflammation and tissue destruction [138]. The ability of natural bioactive compounds to modulate the immune system at a molecular level represents a novel and exciting approach to periodontal therapy [28]. These compounds can shift the immune balance, enhance tissue repair, and stabilize microbial communities, all of which are essential for restoring periodontal health [28].

### 5.1. Shifting the Immune Response: From Pro-Inflammatory to Anti-Inflammatory Profiles

In periodontal disease, the host’s immune response is often skewed towards a pro-inflammatory Th1/Th17 profile, characterized by the production of cytokines like IL-1β, TNF-α, and IL-17, which drive inflammation and tissue destruction [139]. Th17 cells, in particular, play a critical role in promoting osteoclastogenesis through the expression of RANKL, leading to significant periodontal bone loss. Recent research has shown that natural bioactive compounds can modulate this destructive immune profile, promoting a shift towards a Th2/Treg (regulatory T cell) profile, which encourages anti-inflammatory and tissue-protective responses [139].

Curcumin has demonstrated significant immunomodulatory effects by reducing Th17 cell activity while promoting the expansion of regulatory T cells (Tregs). Tregs, in turn, secrete anti-inflammatory cytokines like IL-10 and TGF-β, which inhibit the production of pro-inflammatory cytokines and suppress osteoclast activation [140,141]. Curcumin’s ability to downregulate RANKL expression in periodontal tissues leads to a decrease in osteoclast-mediated bone resorption, effectively reducing bone loss. Additionally, curcumin inhibits NF-κB activation in macrophages and dendritic cells, further diminishing the production of pro-inflammatory mediators and contributing to the restoration of immune balance [140].

Similarly, resveratrol exerts its immunomodulatory effects by suppressing Th1 and Th17 differentiation and enhancing Treg activity [142]. It reduces the production of IL-17 by Th17 cells and IFN-γ by Th1 cells while promoting the secretion of IL-10 by Tregs [143]. In experimental models of periodontal disease, resveratrol treatment has been linked to reduced inflammatory cell infiltration and lower levels of bone loss, underscoring its potential to shift the immune response away from a destructive profile and toward a more protective, anti-inflammatory state [144].

Baicalin, a flavonoid known for its potent immunomodulatory properties, inhibits Th17 differentiation and reduces the production of IL-17, thus limiting the inflammatory response associated with periodontal disease [145]. Baicalin also enhances Treg activity, increasing the expression of Foxp3, a key transcription factor necessary for Treg function [146]. Through these mechanisms, baicalin helps to restore immune homeostasis, reduce inflammation, and protect against tissue destruction in periodontal disease. These natural compounds demonstrate significant potential in modulating the immune response to prevent the progression of periodontal damage [147].

Oleocanthal, a phenolic compound found in extra virgin olive oil, has shown potential as a bioactive agent in periodontal therapy due to its potent anti-inflammatory and antimicrobial properties. Its mechanism of action is believed to center around the inhibition of pro-inflammatory pathways, particularly the cyclooxygenase (COX) enzymes COX-1 and COX-2, which are responsible for producing pro-inflammatory prostaglandins [148,149]. By blocking these enzymes, oleocanthal reduces the inflammatory response in periodontal tissues, thereby mitigating the tissue destruction associated with periodontitis [149].

### 5.2. Enhancement of Efferocytosis and Promotion of Tissue Healing

Efferocytosis is the crucial process by which phagocytes, such as macrophages, clear apoptotic cells, preventing the release of harmful intracellular contents that could exacerbate inflammation and tissue damage [150]. Neutrophils play a significant role in fighting bacterial infections in periodontitis, but their excessive recruitment and activity can lead to tissue damage and the release of pro-inflammatory mediators [151]. As these neutrophils die, they must be efficiently cleared through efferocytosis by macrophages to prevent the accumulation of necrotic debris and further inflammation. Impaired efferocytosis in periodontitis leads to a buildup of apoptotic neutrophils, which can undergo secondary necrosis, releasing toxic cellular contents that perpetuate inflammation and exacerbate tissue destruction [152]. Impaired or delayed efferocytosis in periodontitis leads to the buildup of apoptotic neutrophils, which can undergo secondary necrosis, releasing toxic cellular contents like proteases, reactive oxygen species (ROS), and inflammatory cytokines [153]. Inflammatory cytokines, such as IL-1β, TNF-α, and IL-6, continue to be produced, recruiting more immune cells and creating a cycle of chronic inflammation [154].

In periodontitis, targeting the pathways that regulate efferocytosis—such as the MER receptor tyrosine kinase (MerTK) and Gas6 signaling—could be a therapeutic strategy to resolve chronic inflammation, limit tissue destruction, and promote periodontal regeneration [155].

Resveratrol has been shown to enhance efferocytosis by increasing the expression of MER receptor tyrosine kinase and Gas6, two important regulators of phagocytic activity [156]. By promoting the clearance of apoptotic cells, resveratrol helps reduce the persistence of inflammation and accelerates tissue repair. Additionally, resveratrol stimulates the production of anti-inflammatory lipid mediators, such as resolvins and lipoxins, which are actively involved in resolving inflammation and fostering tissue healing, further emphasizing its therapeutic potential in periodontal disease [157].

Similarly, curcumin enhances efferocytosis by upregulating the expression of CD36, a scavenger receptor that plays a critical role in the recognition and engulfment of apoptotic cells [158]. By facilitating the clearance of neutrophils and other inflammatory cells, curcumin reduces the levels of pro-inflammatory mediators in periodontal tissues. Beyond its role in resolving inflammation, curcumin also promotes tissue regeneration by inducing fibroblast proliferation and collagen synthesis, both of which are essential for restoring periodontal connective tissue integrity [159,160].

Berberine enhances efferocytosis through the activation of AMPK signaling in macrophages, increasing their capacity for phagocytosis [161].

In addition to boosting efferocytosis, berberine inhibits the release of pro-inflammatory cytokines like IL-6 and TNF-α from neutrophils and macrophages, helping to resolve inflammation more effectively. This dual action of enhancing apoptotic cell clearance and reducing pro-inflammatory cytokine production makes berberine a promising candidate for promoting tissue healing in periodontal disease [162].

### 5.3. Impact and Mechanisms of Bioactive Compounds on Osteoclastogenesis, Osteoblastogenesis, and Bone Remodeling

Bone remodeling is a dynamic process that preserves skeletal integrity by maintaining a balance between bone formation (osteoblastogenesis) and bone resorption (osteoclastogenesis) [163]. Flavonoids and polyphenols, including resveratrol, curcumin, and quercetin, exhibit osteoprotective properties by regulating key signaling pathways involved in bone metabolism [163]. Given their ability to modulate these mechanisms, they also hold promise as potential therapeutic agents in the treatment of periodontitis [163].

Quercetin, exerts its osteogenic effects by enhancing the expression of osteogenic markers such as BMP-2, RUNX2, and osteocalcin, thereby promoting osteoblast differentiation and bone matrix formation [164]. It also activates the Wnt/β-catenin signaling pathway, which is essential for bone regeneration. In contrast, quercetin suppresses osteoclastogenesis by downregulating the RANKL/NF-κB signaling pathway and reducing the expression of osteoclast differentiation factors such as c-Fos and NFATc1, which are crucial for osteoclast activation. This inhibitory effect on osteoclastogenesis prevents excessive alveolar bone resorption in periodontitis. Additionally, quercetin plays a crucial role in bone remodeling by maintaining the balance between bone formation and resorption, reducing oxidative stress, and exerting antimicrobial effects against periodontal pathogens, thereby supporting overall periodontal health [164].

Resveratrol significantly influences osteoblastogenesis by activating SIRT1, which enhances osteoblast differentiation and proliferation while protecting bone cells from oxidative stress-induced apoptosis. Its osteogenic effects are further mediated through the activation of the BMP-2/Smad and Wnt/β-catenin pathways, which are essential for bone regeneration [165]. In contrast, resveratrol inhibits osteoclastogenesis by downregulating the NF-κB and MAPK signaling pathways, thereby reducing RANKL-induced osteoclast differentiation and function. This anti-resorptive effect is particularly beneficial in preventing periodontal bone loss. Furthermore, resveratrol contributes to bone remodeling by reducing inflammatory cytokines such as TNF-α, IL-6, and IL-1β, which play a central role in periodontitis-associated bone destruction. Its antioxidant properties also mitigate oxidative stress in the periodontium, further supporting bone homeostasis and periodontal tissue regeneration [165].

**Figure 7:**
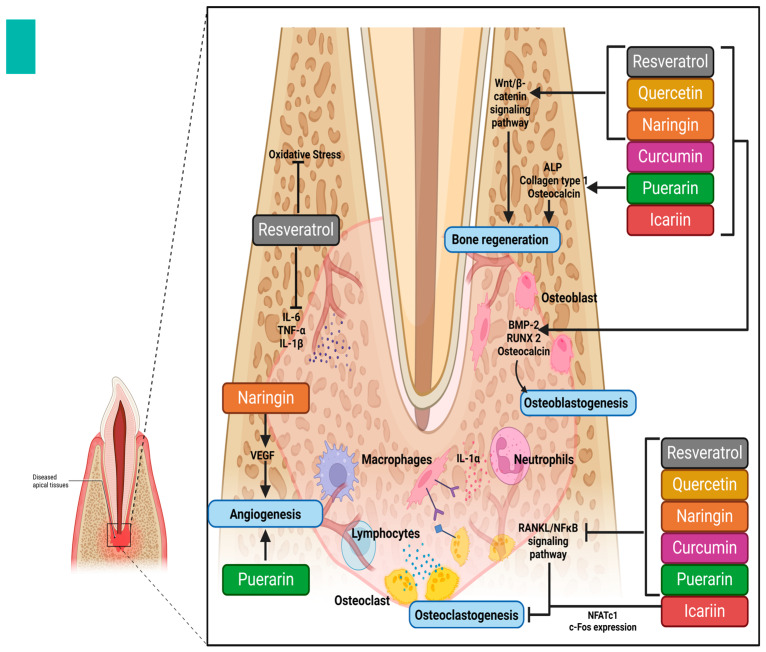
Schematic representation of the effects of bioactive compounds on periodontal bone remodeling. Resveratrol, quercetin, naringin, curcumin, puerarin, and icariin modulate osteoclastogenesis and osteoblastogenesis through pathways such as NF-κB, RANKL, and Wnt/β-catenin. Resveratrol and naringin promote angiogenesis, while puerarin supports vascular endothelial growth factor (VEGF) expression. These compounds collectively enhance bone regeneration and periodontal healing by reducing inflammation and oxidative stress. Created with BioRender.com.

Curcumin promotes osteoblastogenesis by stimulating RUNX2 and osterix expression, both of which are key transcription factors involved in osteogenic differentiation. It enhances ALP activity and increases the deposition of bone matrix proteins such as collagen type I and osteocalcin, making it an effective promoter of bone formation [164]. Simultaneously, curcumin inhibits osteoclastogenesis by suppressing RANKL-mediated NF-κB activation, leading to reduced expression of osteoclast markers such as TRAP and cathepsin K. This results in decreased bone resorption, which is critical in the management of periodontitis-associated bone loss [164]. Additionally, curcumin plays a crucial role in bone remodeling by modulating inflammatory mediators and reducing oxidative stress, both of which contribute to periodontal destruction. Its potent anti-inflammatory properties help suppress periodontal inflammation, thereby protecting alveolar bone and periodontal tissues from degradation [164] (Figure 7).

## 6. Clinical Evidence of Bioactive Compounds in Periodontal Disease Management

Several studies have highlighted the therapeutic potential of natural bioactive compounds, such as curcumin, resveratrol, EGCG (epigallocatechin gallate), and berberine, in managing periodontal disease [28,30]. Although clinical trials remain limited, available evidence suggests that these compounds can enhance periodontal health when used as adjuncts to conventional treatments, such as scaling and root planing (SRP) [166,167,168] (Table 2). For instance, a clinical trial evaluating curcumin gel in combination with SRP demonstrated a significant reduction in plaque index (PI), probing depth (PD), and clinical attachment loss (CAL) (*p* ≤ 0.05) after six weeks, compared to SRP alone [169,170]. Additionally, curcumin treatment led to a notable decrease in procalcitonin (PCT), an inflammatory biomarker, underscoring its anti-inflammatory properties [171]. Similarly, EGCG has been shown to inhibit biofilm formation by up to 80% in *Porphyromonas gingivalis* colonies, highlighting its antimicrobial potential in periodontal therapy [172]. Moreover, resveratrol and quercetin have been reported to suppress osteoclast differentiation by 30–50% in vitro, reinforcing their role in bone remodeling and periodontal regeneration [173,174].

A tri-ketonic phenylaminocarbonyl curcumin analog CMC 2.24, has shown promising results as a pleiotropic matrix metalloproteinase (MMP) inhibitor with both intracellular and extracellular effects. In diabetic rat models with endotoxin-induced periodontitis, systemic administration of CMC 2.24 significantly inhibited alveolar bone loss while also attenuating both local and systemic inflammation. One of its key advantages is its ability to selectively reduce pathologically excessive levels of inducible MMPs—which contribute to periodontal tissue degradation—while preserving constitutive MMPs that are essential for physiological connective tissue turnover [181].

Beyond its periodontal benefits, CMC 2.24 has demonstrated favorable effects on extra-oral connective tissues, skin, and skeletal bone, suggesting potential systemic applications in conditions characterized by inflammation-driven connective tissue destruction. By mitigating hyperglycemia- and bacteria-induced tissue damage, this curcumin derivative represents a novel therapeutic approach that not only preserves periodontal integrity but also exerts systemic protective effects [181].

Despite their promising benefits, bioactive compounds face several challenges in clinical application, primarily due to issues related to bioavailability and stability in the oral environment. For example, both curcumin and resveratrol exhibit low oral bioavailability, which limits their therapeutic potential. Similarly, catechins (EGCG) are unstable in the oral environment, reducing their effectiveness. Berberine, although effective in preclinical studies, has yet to be tested in large-scale human trials [182,183,184].

Addressing these challenges, such as through advanced delivery systems like nanoparticles, is crucial for their successful translation into clinical practice. These challenges are further detailed in Table 3.

## 7. Exploring Novel Molecular Mechanisms of Bioactive Compounds in Periodontal Therapy: A Forward-Thinking Approach

### 7.1. Curcumin and Epigenetic Modulation Beyond HDACs

While curcumin’s role as a histone deacetylase (HDAC) inhibitor is well established, emerging studies in oncology and neurobiology suggest that curcumin may influence non-coding RNAs (ncRNAs), including microRNAs (miRNAs) and long non-coding RNAs (lncRNAs) [185,186].

These ncRNAs are critical for post-transcriptional regulation and have been shown to modulate immune responses and inflammation [186].

Curcumin could regulate miR-146a, miRNA involved in immune suppression, leading to a novel mechanism for reducing inflammation in periodontal disease. Moreover, curcumin might alter lncRNA-ANRIL, which has been implicated in regulating inflammation and atherosclerosis. Investigating whether curcumin affects these ncRNAs in the context of periodontal disease could open new avenues for exploring how curcumin orchestrates gene silencing beyond traditional epigenetic marks [187].

### 7.2. Resveratrol and Mitochondrial Biogenesis

Although resveratrol’s effect on the SIRT1 pathway and its anti-inflammatory properties are well-known, more recent data suggest that resveratrol can modulate mitochondrial biogenesis through the PGC-1α (peroxisome proliferator-activated receptor gamma coactivator-1 alpha) pathway [165,188]. Mitochondria play an essential role not just in energy metabolism but also in regulating inflammation and apoptosis. Resveratrol may enhance mitochondrial quality control, preventing excessive ROS production in periodontal tissues and limiting oxidative stress. Additionally, mitochondrial dysfunction has been linked to aging-related diseases, and by targeting mitochondrial biogenesis, resveratrol might not only mitigate inflammation but also promote cellular longevity in gingival and periodontal ligament cells [189,190].

### 7.3. Quercetin and Autophagy Regulation

Quercetin’s anti-inflammatory properties are well-established, but recent evidence hints at its ability to modulate autophagy, a process that clears damaged proteins and organelles, thus maintaining cellular homeostasis [48]. Autophagy dysregulation is associated with both chronic inflammation and periodontal disease [191,192]. By activating autophagy-related proteins such as Beclin-1 and LC3-II, quercetin may help in clearing damaged or stressed cells in periodontal tissues, thus reducing persistent inflammation and promoting tissue healing. In this context, quercetin could act as a dual regulator of inflammation and cellular clearance, especially in oxidative stress-driven damage in the periodontal environment [193].

### 7.4. Catechins and Gut-Oral Microbiome Crosstalk

Green tea catechins, such as EGCG, are known for their antimicrobial properties against periodontal pathogens [110,113]. However, novel research suggests that catechins could play a significant role in modulating the gut-oral microbiome axis, a new area of research gaining traction in the fields of systemic diseases and oral health [194].

Catechins may influence gut microbiota composition by promoting the growth of butyrate-producing bacteria, which have anti-inflammatory effects on systemic immunity [195].

The production of short-chain fatty acids (SCFAs) by gut microbiota might exert systemic anti-inflammatory effects, indirectly benefiting periodontal health. EGCG could bridge the oral and gut microbiomes, influencing systemic inflammation and enhancing periodontal disease resolution through an intricate gut-oral microbiome interaction [196].

### 7.5. Baicalin and Immune Metabolism

Baicalin has demonstrated antimicrobial and anti-inflammatory properties, but its ability to modulate immune metabolism is an exciting new frontier [197]. Immune cells, such as macrophages, undergo metabolic reprogramming (termed immunometabolism) during inflammation. Baicalin might alter metabolic pathways in macrophages, shifting them from an inflammatory (glycolysis-driven) state to a reparative (oxidative phosphorylation-driven) state [198]. This could modulate the mTOR pathway, which controls cell metabolism, promoting the transition from pro-inflammatory M1 macrophages to anti-inflammatory M2 macrophages in periodontal lesions. By reprogramming immune cell metabolism, baicalin could help maintain immune homeostasis and enhance tissue regeneration in the periodontal microenvironment [199].

### 7.6. Carvacrol and Quorum Sensing Inhibition in Multi-Species Biofilms

Carvacrol’s ability to inhibit quorum sensing in bacterial biofilms is well recognized, but emerging studies are exploring the interaction between polymicrobial biofilms and host responses [200]. Multi-species biofilms, particularly those involving *P. gingivalis*, *T. forsythia*, and *F. nucleatum*, exhibit sophisticated inter-bacterial communication that promotes virulence and resistance to treatment [201].

Carvacrol could serve as a specific inhibitor of multi-species quorum sensing, targeting cross-species bacterial signaling molecules like autoinducer-2 (AI-2), thus reducing biofilm resistance to both antimicrobial agents and host immune clearance. Carvacrol could also enhance the efficacy of probiotics, promoting a balanced oral microbiome and reducing the virulence of dysbiotic communities [200].

### 7.7. β-Caryophyllene and Endocannabinoid System Modulation in Inflammation

β-Caryophyllene’s interaction with the CB2 receptor in the endocannabinoid system is of growing interest for its ability to modulate inflammation without psychoactive effects [202]. CB2 receptor activation has been associated with the suppression of inflammatory cytokines and enhanced tissue repair [203]. Exosomes carrying β-caryophyllene could be explored as a targeted delivery system for periodontal tissues, allowing for precise control of inflammation at the local level [204]. Moreover, β-caryophyllene could synergize with endogenous cannabinoids, enhancing the natural resolution of inflammation and promoting tissue regeneration in advanced periodontal lesions [205].

### 7.8. Berberine and MicroRNA-Based Modulation

Recent studies suggest that berberine may exert its anti-inflammatory effects by modulating the expression of microRNAs (miRNAs) that control key inflammatory pathways [206]. Specifically, berberine could downregulate miR-21, a microRNA linked to inflammation, fibrosis, and ECM degradation in periodontal tissues. Through miRNA-based modulation, berberine might help reduce periodontal tissue destruction at a genetic level, offering long-term protective effects [207,208].

Additionally, berberine could influence exosome-mediated intercellular communication, promoting anti-inflammatory signals in gingival epithelial cells [209].

### 7.9. Essential Oils and Synergistic Effects in Nanotechnology-Based Delivery Systems

While essential oils like thymol and eucalyptol have known antimicrobial properties, their potential is greatly expanded when integrated into nanoparticle delivery systems. These nano-formulations can enhance the penetration of essential oils into biofilms and periodontal tissues, ensuring sustained release and prolonged efficacy [210].

Nanoencapsulation of essential oils could also reduce volatility and ensure targeted antimicrobial action, allowing for the disruption of biofilms at deep periodontal sites that are difficult to reach with conventional treatments [211].

### 7.10. Gum Arabic and Modulation of the Oral-Immune Interface

**Figure 8:**
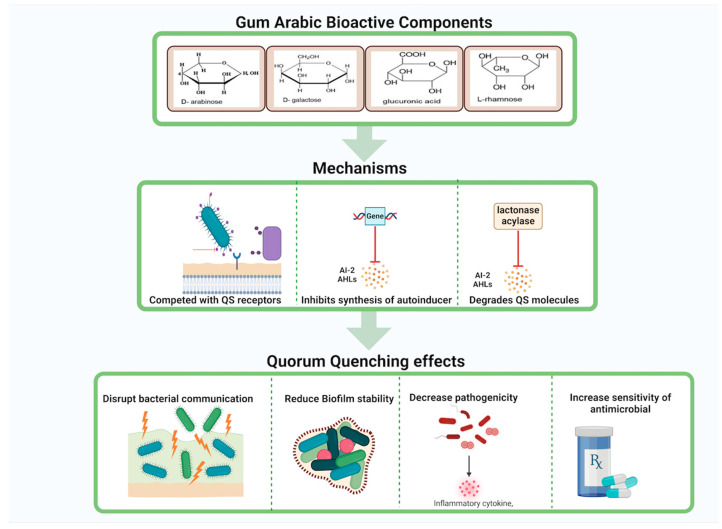
Highlights the bioactive components of Gum Arabic and their role in quorum quenching mechanisms. The top section presents the chemical structures of key Gum Arabic compounds. The middle section illustrates mechanisms such as competing with quorum sensing (QS) receptors, inhibiting oxidoreductase synthesis, and degrading QS molecules via lactonase activity. The bottom section outlines the resulting quorum quenching effects, including disrupted bacterial communication, reduced biofilm stability, decreased pathogenicity through inflammatory cytokine inhibition, and increased antimicrobial sensitivity. Created with BioRender.com.

Gum Arabic’s prebiotic properties and ability to modulate the oral microbiome are already acknowledged, but a more advanced concept involves its role in modulating the oral-immune interface [31]. Gum Arabic may influence mucosal immunity by interacting with dendritic cells and modulating T regulatory (Treg) cell responses. By enhancing mucosal immune tolerance, Gum Arabic could reduce the risk of chronic inflammation and promote a balanced immune response. Moreover, Gum Arabic could be used as a biofilm-dispersing agent, working synergistically with other antimicrobials to break down established biofilms and enhance the efficacy of periodontal therapies [196] (Figure 8).

### 7.11. Potential Cytotoxicity of High Doses of Curcumin, EGCG, Resveratrol, Baicalin, and Quercetin

While bioactive compounds such as curcumin, EGCG, and resveratrol offer significant therapeutic benefits, their cytotoxic effects at high doses raise concerns regarding their safe application.

Curcumin has a well-established safety profile and is recognized as GRAS (Generally Recognized as Safe) by the FDA, with human studies indicating tolerance at doses up to 8 g/day [212]. However, in vitro and animal studies have reported dose-dependent toxicity, including DNA fragmentation, hepatotoxicity, and gastrointestinal disturbances at high concentrations. Acute toxicity studies in humans found mild, non-serious side effects at doses above 10–12 g/day [213]. Long-term animal studies linked extremely high doses to liver toxicity, gastrointestinal issues, and carcinogenic potential in rodents, but no significant adverse effects were observed in clinical trials with doses up to 8 g/day. Overall, curcumin remains safe at moderate doses, though higher doses require caution due to potential cytotoxic effects [214]. A recent study has shown that EGCG exhibits acute and subacute toxicity, particularly through its effects on mitochondrial dysfunction in primary astrocytes. The mechanism of EGCG-induced toxicity is associated with Ca^2+^ overloading, which occurs via voltage-gated calcium channels (VGCCs) and mitochondrial Ca^2+^ uniporter (MCU) [215]. Resveratrol, a phytochemical with potential health benefits, has also been reported to exhibit cytotoxic effects, primarily through the inhibition of the oxidative phosphorylation (OXPHOS) pathway. Its toxicity appears to be energy-dependent, whereas, in low-energy conditions, resveratrol can exacerbate ATP depletion, leading to cell death. Conversely, when cells have high energy availability, resveratrol-induced OXPHOS inhibition may create a controlled low-energy state, mimicking caloric restriction benefits. This dual effect suggests a complex relationship between caloric intake and resveratrol’s impact on cellular metabolism, with potential applications in cancer therapy [216,217]. High doses (≥50 μM) of EGCG lead to mitochondrial permeability transition pore (mPTP) opening, membrane depolarization, increased reactive oxygen species (ROS), and cytochrome c release, ultimately triggering apoptosis. More apoptotic cells were observed at 50 μM EGCG compared to lower doses (1 μM EGCG), indicating dose-dependent cytotoxicity. These findings suggest that high-dose EGCG may be toxic to astrocytes by targeting mitochondria via calcium dysregulation, expanding our understanding of its potential neurotoxicity and safety considerations [218,219].

## 8. Gaps in Current Research

Despite the promising preclinical and early clinical findings, several significant gaps remain in the research on bioactive compounds in periodontal therapy:

- Lack of Large-Scale Clinical Trials: Most clinical studies to date have been small and short-term. Larger, randomized controlled trials (RCTs) with longer follow-up periods are needed to establish the efficacy and safety of bioactive compounds as adjuncts to periodontal therapy.
- Bioavailability Issues: A common challenge for many bioactive compounds, including curcumin, resveratrol, and catechins, is their poor bioavailability in oral tissues. This limits their clinical efficacy, as only a small fraction of the administered dose reaches the target site. Strategies to improve bioavailability, such as nanoparticle delivery systems, encapsulation techniques, and sustained-release formulations, need to be further explored.
- Heterogeneity in Study Designs: There is a lack of standardization in clinical trial designs, including variations in dosage, duration, and forms of bioactive compound administration. This makes it difficult to compare results across studies and draw definitive conclusions about efficacy.
- Mechanism of Action in Humans: While there is substantial preclinical evidence on the molecular mechanisms of bioactive compounds in periodontal tissues, these mechanisms have not been fully validated in human studies. Future research should focus on identifying specific molecular targets and signaling pathways in human clinical settings.

## 9. Future Directions: Addressing the Gaps

To overcome the existing challenges and fully harness the potential of bioactive compounds in periodontal therapy, the following strategies should be pursued:

- Advanced Delivery Systems: Nanoparticles, liposomes, and hydrogels offer promising solutions for improving the bioavailability of bioactive compounds. For example, curcumin-loaded nanoparticles have been shown to improve curcumin’s stability, enhance its penetration into biofilms, and provide sustained release in periodontal pockets. Similar formulations for other bioactives like resveratrol and EGCG are being explored to optimize their delivery and maximize therapeutic efficacy.
- Combination Therapies: Pairing bioactive compounds with conventional periodontal treatments, such as antibiotics or mechanical debridement, may offer synergistic effects. For instance, combining curcumin or resveratrol with low-dose antibiotics could enhance antimicrobial efficacy while reducing the risk of antibiotic resistance. Combination therapies should be rigorously tested in well-designed clinical trials.
- Personalized Periodontal Care: Future research should focus on the development of personalized therapies that take into account the patient’s genetic, microbiome, and immunological profiles. By tailoring bioactive compound use to the individual patient, clinicians could maximize therapeutic outcomes. For example, genetic markers of inflammation or bone resorption could guide the selection of specific bioactive compounds that target these pathways.
- Long-Term Studies: It is essential to conduct long-term follow-up studies to evaluate the sustained effects of bioactive compounds in preventing disease recurrence. Periodontal disease is chronic, and the long-term stability of treatment outcomes is crucial for patient care.
- Despite promising findings on the therapeutic potential of bioactive compounds in periodontal disease, most preclinical and clinical studies do not differentiate between mild, moderate, and severe periodontitis when evaluating their efficacy. Future research should aim to stratify patients based on disease severity to determine whether bioactive compounds exert differential effects at various stages of periodontal destruction.
- Exploration of Synergistic Combinations of Bioactive Compounds: Natural bioactive compounds often exhibit complementary mechanisms of action. Future studies should explore combinations of compounds, such as curcumin with EGCG or berberine with resveratrol, to evaluate whether their synergistic effects can further enhance periodontal regeneration and inflammation control.

## 10. Conclusions

Natural bioactive compounds represent a transformative potential in the management of periodontal disease, offering a multi-faceted approach that targets both microbial pathogens and host immune responses at an advanced molecular level. The unique properties of these compounds—including their anti-inflammatory, antimicrobial, antioxidant, and tissue-regenerative effects—enable them to address the complex etiopathogenesis of periodontal disease, which involves chronic inflammation, microbial dysbiosis, oxidative stress, and tissue destruction.

Preclinical studies have shown that compounds such as curcumin, resveratrol, EGCG, baicalin, berberine, and essential oils exhibit potent effects in reducing bacterial colonization, inhibiting biofilm formation, and modulating key inflammatory pathways like NF-κB, MAPK, and JAK/STAT. These compounds also promote tissue healing by enhancing efferocytosis, stimulating collagen synthesis, and promoting osteoblast activity, all of which are critical for the preservation of periodontal tissues. Furthermore, some compounds exhibit epigenetic modulating properties, offering long-term benefits through gene expression regulation in periodontal tissues.

Despite promising findings from in vitro, in vivo, and early clinical trials, significant gaps remain in fully realizing the therapeutic potential of bioactive compounds in periodontal care. Key challenges include their poor bioavailability, short retention in the oral cavity, and limited large-scale human clinical trials. Addressing these issues through advanced delivery systems, such as nanoparticles, hydrogels, and sustained-release formulations, could greatly improve the efficacy of bioactive compounds in clinical settings. Additionally, the development of combination therapies, personalized periodontal treatments, and long-term follow-up studies will be critical for establishing these compounds as integral components of periodontal therapy.

The future of periodontal treatment may well lie in the integration of natural bioactive compounds with conventional therapies, as they offer safer, more holistic, and potentially more effective approaches to managing this chronic and widespread disease. As research continues to evolve, bioactive compounds could become essential adjuncts in promoting not only the treatment of periodontal disease but also its prevention, thus significantly improving patient outcomes and reducing the global burden of periodontal disease.

## Figures and Tables

**Table 1:** Summarizes the key actions, used forms, expected side effects, and specific molecular targets of various bioactive compounds that can be applied as adjuncts in the treatment of periodontal disease. Each compound offers unique benefits in modulating inflammation, controlling microbial activity, and promoting tissue regeneration.

Compound	Action	Used Form	Expected Side Effects	Specific Target
Curcumin	Anti-inflammatory, antioxidant, antimicrobial	Topical gel	Mild irritation or allergic reactions	NF-κB, COX-2, MMP-9
Resveratrol	Anti-inflammatory, antioxidant	Mouthwash or gel	Rare gastrointestinal upset, allergic reaction	NF-κB, Nrf2
Quercetin	Anti-inflammatory, antioxidant, MMP inhibition	Mouthwash or topical gel	Minimal, possible allergic reactions	MAPK, NF-κB, MMPs
Catechins (EGCG)	Antimicrobial, anti-inflammatory, antioxidant	Mouthwash or chewing gum	Possible mild irritation	MMPs, NF-κB, periodontal pathogens
Baicalin	Anti-inflammatory, antimicrobial	Mouth rinse or topical gel	Rare allergic reactions	MAPK, periodontal pathogens
Carvacrol	Antimicrobial, biofilm disruption	Mouthwash or essential oil gel	Oral irritation, burning sensation	Bacterial cell membranes, quorum sensing
β-Caryophyllene	Anti-inflammatory, antimicrobial, antioxidant	Topical gel or mouthwash	Minimal, potential oral irritation	CB2 receptor, microbial cell walls
Berberine	Antimicrobial, anti-inflammatory	Gel or toothpaste	Gastrointestinal upset in high doses	TNF-α, IL-6, periodontal pathogens
Essential Oils (Thymol, Eucalyptol)	Antimicrobial, anti-inflammatory	Mouthwash	Oral irritation, taste alteration	Bacterial cell membranes, pro-inflammatory cytokines
Antimicrobial Peptides (AMPs)	Antimicrobial, immune modulation	Topical gel or mouthwash	Minimal, rare allergic reactions	Bacterial membranes, immune cells

**Table 2:** Clinical studies of bioactive compounds in periodontitis.

Compound	Study Type	Findings
Curcumin	Clinical Trials	Reduces PD, CAL, and BOP when used as an adjunct to SRP [175,176]
Resveratrol	Clinical Trials	Reduces gingival inflammation and probing depth in patients treated with resveratrol gels [177].
EGCG (Green Tea)	Clinical Trials	Reduces plaque index, probing depth, and gingival inflammation in green tea-based mouthwash and gel trials [178,179].
Berberine	Clinical Trials	Improves gingival health and reduces gingival index and pocket depth when used with SRP [180].

**Table 3:** Challenges of bioactive compounds in periodontitis.

Compound	Challenges
Curcumin	Poor bioavailability in oral tissues; nanoparticle formulations and hydrogels are being developed to overcome this.
Resveratrol	Low oral bioavailability and rapid metabolism; advanced delivery mechanisms are being explored.
Catechins (EGCG)	Instability of catechins in the oral environment; new formulations are needed to preserve bioactivity.
Berberine	Limited large-scale human trials; bioavailability needs improvement through novel delivery methods.

## Data Availability

No new data were created or analyzed in this study.

## References

[1] Kinane D.F., Stathopoulou P.G., Papapanou P.N. (2017). Periodontal diseases. Nat. Rev. Dis. Primers.

[2] Abdulkareem A.A., Al-Taweel F.B., Al-Sharqi A.J.B., Gul S.S., Sha A., Chapple I.L.C. (2023). Current concepts in the pathogenesis of periodontitis: From symbiosis to dysbiosis. J. Oral Microbiol..

[3] Hashimoto M., Ogawa S., Asai Y., Takai Y., Ogawa T. (2003). Binding of *Porphyromonas gingivalis* fimbriae to *Treponema denticola* dentilisin. FEMS Microbiol. Lett..

[4] Xu W., Zhou W., Wang H., Liang S. (2020). Roles of *Porphyromonas gingivalis* and its virulence factors in periodontitis. Adv. Protein Chem. Struct. Biol..

[5] Dias I.H.K., Marshall L., Lambert P.A., Chapple I.L.C., Matthews J.B., Griffiths H.R. (2008). Gingipains from *Porphyromonas gingivalis* increase the chemotactic and respiratory burst-priming properties of the 77-amino-acid interleukin-8 variant. Infect. Immun..

[6] Huang R., Li M., Gregory R.L. (2011). Bacterial interactions in dental biofilm. Virulence.

[7] Acosta I.C., Alonzo F. (2023). The Intersection between Bacterial Metabolism and Innate Immunity. J. Innate Immun..

[8] Hashim N., Babiker R., Mohammed R., Rehman M.M., Chaitanya N.C., Gobara B. (2024). NLRP3 Inflammasome in Autoinflammatory Diseases and Periodontitis: Advances in the Management. J. Pharm. Bioallied Sci..

[9] Scott D.A., Krauss J. (2012). Neutrophils in periodontal inflammation. Periodontal Dis..

[10] Sima C., Viniegra A., Glogauer M. (2019). Macrophage immunomodulation in chronic osteolytic diseases-the case of periodontitis. J. Leukoc. Biol..

[11] Zhou P., Zheng T., Zhao B. (2022). Cytokine-mediated immunomodulation of osteoclastogenesis. Bone.

[12] Luchian I., Goriuc A., Sandu D., Covasa M. (2022). The Role of Matrix Metalloproteinases (MMP-8, MMP-9, MMP-13) in Periodontal and Peri-Implant Pathological Processes. Int. J. Mol. Sci..

[13] Shang J., Liu H., Zheng Y., Zhang Z. (2023). Role of oxidative stress in the relationship between periodontitis and systemic diseases. Front. Physiol..

[14] Tariq M., Iqbal Z., Ali J., Baboota S., Talegaonkar S., Ahmad Z., Sahni J.K. (2012). Treatment modalities and evaluation models for periodontitis. Int. J. Pharm. Investig..

[15] Sinha S., Sonoo P.R., Siddhartha R., Singh S.K., Singh A. (2021). Effect of Conventional Periodontal Treatment (Scaling and Root Planing) on Type-2 Diabetic Patient with Moderate Generalized Chronic Periodontitis: A Clinical Study. J. Pharm. Bioallied Sci..

[16] Socransky S.S., Haffajee A.D. (2002). Dental biofilms: Difficult therapeutic targets. Periodontology 2000.

[17] Cobb C.M., Sottosanti J.S. (2021). A re-evaluation of scaling and root planing. J. Periodontol..

[18] Khattri S., Kumbargere Nagraj S., Arora A., Eachempati P., Kusum C.K., Bhat K.G., Johnson T.M., Lodi G. (2020). Adjunctive systemic antimicrobials for the non-surgical treatment of periodontitis. Cochrane Database Syst. Rev..

[19] Llor C., Bjerrum L. (2014). Antimicrobial resistance: Risk associated with antibiotic overuse and initiatives to reduce the problem. Ther. Adv. Drug Saf..

[20] Kesavelu D., Jog P. (2023). Current understanding of antibiotic-associated dysbiosis and approaches for its management. Ther. Adv. Infect. Dis..

[21] Wang H., Greenwell H. (2001). Surgical periodontal therapy. Periodontology 2000.

[22] Wang G., Yuan F., Ying W., Xu J. (2023). The effects of different regenerative technologies and materials on wound healing after surgical endodontic therapy: A meta-analysis. Int. Wound J..

[23] Marín-Jaramillo R.A., Agudelo-Suárez A.A. (2022). Factors related to compliance with periodontal disease treatment appointments: A literature review. J. Clin. Exp. Dent..

[24] Huang T.-H., Chen J.-Y., Suo W.-H., Shao W.-R., Huang C.-Y., Li M.-T., Li Y.-Y., Li Y.-H., Liang E.-L., Chen Y.-H. (2024). Unlocking the Future of Periodontal Regeneration: An Interdisciplinary Approach to Tissue Engineering and Advanced Therapeutics. Biomedicines.

[25] Puletic M., Velikic G., Maric D.M., Supic G., Maric D.L., Radovic N., Avramov S., Vojvodic D. (2024). Clinical Efficacy of Extracellular Vesicle Therapy in Periodontitis: Reduced Inflammation and Enhanced Regeneration. Int. J. Mol. Sci..

[26] Iheozor-Ejiofor Z., Middleton P., Esposito M., Glenny A.M. (2017). Treating periodontal disease for preventing adverse birth outcomes in pregnant women. Cochrane Database Syst. Rev..

[27] Păunică I., Giurgiu M., Dumitriu A.S., Păunică S., Pantea Stoian A.M., Martu M.A., Serafinceanu C. (2023). The Bidirectional Relationship between Periodontal Disease and Diabetes Mellitus—A Review. Diagnostics.

[28] Hashim N.T., Babiker R., Rahman M.M., Mohamed R., Priya S.P., Chaitanya N.C., Islam M.S., Gobara B. (2024). Natural Bioactive Compounds in the Management of Periodontal Diseases: A Comprehensive Review. Molecules.

[29] Sorrenti V., Burò I., Consoli V., Vanella L. (2023). Recent Advances in Health Benefits of Bioactive Compounds from Food Wastes and By-Products: Biochemical Aspects. Int. J. Mol. Sci..

[30] McCubrey J.A., Lertpiriyapong K., Steelman L.S., Abrams S.L., Yang L.V., Murata R.M., Rosalen P.L., Scalisi A., Neri L.M., Cocco L. (2017). Effects of resveratrol, curcumin, berberine and other nutraceuticals on aging, cancer development, cancer stem cells and microRNAs. Aging.

[31] Al-Jubori Y., Ahmed N.T.B., Albusaidi R., Madden J., Das S., Sirasanagandla S.R. (2023). The Efficacy of Gum Arabic in Managing Diseases: A Systematic Review of Evidence-Based Clinical Trials. Biomolecules.

[32] Bezerra B., Monajemzadeh S., Silva D., Pirih F.Q. (2022). Modulating the Immune Response in Periodontitis. Front. Dent. Med..

[33] Mazzanti G., Di Giacomo S. (2016). Curcumin and Resveratrol in the Management of Cognitive Disorders: What is the Clinical Evidence?. Molecules.

[34] David A.V.A., Arulmoli R., Parasuraman S. (2016). Overviews of Biological Importance of Quercetin: A Bioactive Flavonoid. Pharmacogn. Rev..

[35] Musial C., Kuban-Jankowska A., Gorska-Ponikowska M. (2020). Beneficial Properties of Green Tea Catechins. Int. J. Mol. Sci..

[36] Yin B., Li W., Qin H., Yun J., Sun X. (2021). The Use of Chinese Skullcap (*Scutellaria baicalensis*) and Its Extracts for Sustainable Animal Production. Animals.

[37] Mączka W., Twardawska M., Grabarczyk M., Wińska K. (2023). Carvacrol—A Natural Phenolic Compound with Antimicrobial Properties. Antibiotics.

[38] Tsigoriyna L., Sango C., Batovska D. (2024). An Update on Microbial Biosynthesis of β-Caryophyllene, a Sesquiterpene with Multi-Pharmacological Properties. Fermentation.

[39] Kowalczyk A., Przychodna M., Sopata S., Bodalska A., Fecka I. (2020). Thymol and Thyme Essential Oil-New Insights into Selected Therapeutic Applications. Molecules.

[40] Satchanska G., Davidova S., Gergova A. (2024). Diversity and Mechanisms of Action of Plant, Animal, and Human Antimicrobial Peptides. Antibiotics.

[41] Liu T., Zhang L., Joo D., Sun S.C. (2017). NF-κB signaling in inflammation. Signal Transduct. Target. Ther..

[42] Oeckinghaus A., Ghosh S. (2009). The NF-kappaB family of transcription factors and its regulation. Cold Spring Harb. Perspect Biol..

[43] Guo Q., Jin Y., Chen X., Ye X., Shen X., Lin M., Zeng C., Zhou T., Zhang J. (2024). NF-κB in biology and targeted therapy: New insights and translational implications. Signal Transduct. Target. Ther..

[44] Al-Qahtani A.A., Alhamlan F.S., Al-Qahtani A.A. (2024). Pro-Inflammatory and Anti-Inflammatory Interleukins in Infectious Diseases: A Comprehensive Review. Trop. Med. Infect. Dis..

[45] Choi Y., Lee M.K., Lim S.Y., Sung S.H., Kim Y.C. (2009). Inhibition of inducible NO synthase, cyclooxygenase-2 and interleukin-1beta by torilin is mediated by mitogen-activated protein kinases in microglial BV2 cells. Br. J. Pharmacol..

[46] Sohn S.I., Priya A., Balasubramaniam B., Muthuramalingam P., Sivasankar C., Selvaraj A., Valliammai A., Jothi R., Pandian S. (2021). Biomedical Applications and Bioavailability of Curcumin—An Updated Overview. Pharmaceutics.

[47] Ye Y., Zhou J. (2023). The protective activity of natural flavonoids against osteoarthritis by targeting NF-κB signaling pathway. Front. Endocrinol..

[48] Aghababaei F., Hadidi M. (2023). Recent Advances in Potential Health Benefits of Quercetin. Pharmaceuticals.

[49] Joo S.Y., Song Y.A., Park Y.L., Myung E., Chung C.Y., Park K.J., Cho S.B., Lee W.S., Kim H.S., Rew J.S. (2012). Epigallocatechin-3-gallate Inhibits LPS-Induced NF-κB and MAPK Signaling Pathways in Bone Marrow-Derived Macrophages. Gut Liver.

[50] Olivera A., Moore T.W., Hu F., Brown A.P., Sun A., Liotta D.C., Snyder J.P., Yoon Y., Shim H., Marcus A.I. (2012). Inhibition of the NF-κB signaling pathway by the curcumin analog, 3,5-Bis(2-pyridinylmethylidene)-4-piperidone (EF31): Anti-inflammatory and anti-cancer properties. Int. Immunopharmacol..

[51] Xu L., Botchway B.O.A., Zhang S., Zhou J., Liu X. (2018). Inhibition of NF-κB Signaling Pathway by Resveratrol Improves Spinal Cord Injury. Front. Neurosci..

[52] Zhao H., Wu L., Yan G., Chen Y., Zhou M., Wu Y., Li Y. (2021). Inflammation and tumor progression: Signaling pathways and targeted intervention. Signal Transduct. Target. Ther..

[53] Ma Q. (2013). Role of nrf2 in oxidative stress and toxicity. Annu. Rev. Pharmacol. Toxicol..

[54] Wang Y., Branicky R., Noë A., Hekimi S. (2018). Superoxide dismutases: Dual roles in controlling ROS damage and regulating ROS signaling. J. Cell Biol..

[55] Ngo V., Duennwald M.L. (2022). Nrf2 and Oxidative Stress: A General Overview of Mechanisms and Implications in Human Disease. Antioxidants.

[56] Zhang L., Xu L.Y., Tang F., Liu D., Zhao X.L., Zhang J.N., Xia J., Wu J.J., Yang Y., Peng C. (2024). New perspectives on the therapeutic potential of quercetin in non-communicable diseases: Targeting Nrf2 to counteract oxidative stress and inflammation. J. Pharm. Anal..

[57] Baird L., Yamamoto M. (2020). The Molecular Mechanisms Regulating the KEAP1-NRF2 Pathway. Mol. Cell. Biol..

[58] Almerich-Silla J.M., Montiel-Company J.M., Pastor S., Serrano F., Puig-Silla M., Dasí F. (2015). Oxidative stress parameters in saliva and its association with periodontal disease and types of bacteria. Dis. Markers.

[59] Ma F., Luo S., Lu C., Jiang X., Chen K., Deng J., Ma S., Li Z. (2022). The role of Nrf2 in periodontal disease by regulating lipid peroxidation, inflammation and apoptosis. Front. Endocrinol..

[60] Shahcheraghi S.H., Salemi F., Peirovi N., Ayatollahi J., Alam W., Khan H., Saso L. (2021). Nrf2 Regulation by Curcumin: Molecular Aspects for Therapeutic Prospects. Molecules.

[61] Xu G., Ma Y., Jin J., Wang X. (2022). Activation of AMPK/p38/Nrf2 is involved in resveratrol alleviating myocardial ischemia-reperfusion injury in diabetic rats as an endogenous antioxidant stress feedback. Ann. Transl. Med..

[62] Mokra D., Joskova M., Mokry J. (2022). Therapeutic Effects of Green Tea Polyphenol (−)-Epigallocatechin-3-Gallate (EGCG) in Relation to Molecular Pathways Controlling Inflammation, Oxidative Stress, and Apoptosis. Int. J. Mol. Sci..

[63] Wong S.K., Chin K.Y., Ima-Nirwana S. (2020). Quercetin as an Agent for Protecting the Bone: A Review of the Current Evidence. Int. J. Mol. Sci..

[64] Lesjak M., Beara I., Simin N., Pintać D., Majkić T., Bekvalac K., Orčić D., Mimica-Dukić N. (2018). Antioxidant and anti-inflammatory activities of quercetin and its de-rivatives. J. Funct. Foods.

[65] Xu D., Hu M.-J., Wang Y.-Q., Cui Y.-L. (2019). Antioxidant Activities of Quercetin and Its Complexes for Medicinal Application. Molecules.

[66] Morris R., Kershaw N.J., Babon J.J. (2018). The molecular details of cytokine signaling via the JAK/STAT pathway. Protein Sci..

[67] Seif F., Khoshmirsafa M., Aazami H., Mohsenzadegan M., Sedighi G., Bahar M. (2017). The role of JAK-STAT signaling pathway and its regulators in the fate of T helper cells. Cell Commun. Signal..

[68] Chimento A., D’amico M., De Luca A., Conforti F.L., Pezzi V., De Amicis F. (2023). Resveratrol, Epigallocatechin Gallate and Curcumin for Cancer Therapy: Challenges from Their Pro-Apoptotic Properties. Life.

[69] Golmohammadi M., Zamanian M.Y., Al-Ani A.M., Jabbar T.L., Kareem A.K., Aghaei Z.H., Tahernia H., Hjazi A., Jissir S.A., Hakimizadeh E. (2024). Targeting STAT3 signaling pathway by curcumin and its analogues for breast cancer: A narrative review. Anim. Models Exp. Med..

[70] Zhang C., Peng Q., Tang Y., Wang C., Wang S., Yu D., Hou S., Wang Y., Zhang L., Lin N. (2024). Resveratrol ameliorates glioblastoma inflammatory response by reducing NLRP3 inflammasome activation through inhibition of the JAK2/STAT3 pathway. J. Cancer Res. Clin. Oncol..

[71] Moens U., Kostenko S., Sveinbjørnsson B. (2013). The Role of Mitogen-Activated Protein Kinase-Activated Protein Kinases (MAPKAPKs) in Inflammation. Genes.

[72] Yu C., Wang D., Yang Z., Wang T. (2022). Pharmacological Effects of Polyphenol Phytochemicals on the Intestinal Inflammation via Targeting TLR4/NF-κB Signaling Pathway. Int. J. Mol. Sci..

[73] Ko S.Y. (2021). Baicalin suppresses lipopolysaccharide-induced matrix metalloproteinase expression: Action via the mitogen-activated protein kinase and nuclear factor κB-related protein signaling pathway. Int. J. Oral Biol..

[74] Meng T., Xiao D., Muhammed A., Deng J., Chen L., He J. (2021). Anti-Inflammatory Action and Mechanisms of Resveratrol. Molecules.

[75] Radzki D., Negri A., Kusiak A., Obuchowski M. (2024). Matrix Metalloproteinases in the Periodontium-Vital in Tissue Turnover and Unfortunate in Periodontitis. Int. J. Mol. Sci..

[76] Cabral-Pacheco G.A., Garza-Veloz I., la Rosa C.C.-D., Ramirez-Acuña J.M., Perez-Romero B.A., Guerrero-Rodriguez J.F., Martinez-Avila N., Martinez-Fierro M.L. (2020). The Roles of Matrix Metalloproteinases and Their Inhibitors in Human Diseases. Int. J. Mol. Sci..

[77] Mogharrabi M., Rahimi H.R., Hasanzadeh S., Dastani M., Kazemi-Oskuee R., Akhlaghi S., Soukhtanloo M. (2020). The effects of nanomicelle of curcumin on the matrix metalloproteinase (MMP-2, 9) activity and expression in patients with coronary artery disease (CAD): A randomized controlled clinical trial. ARYA Atheroscler..

[78] Sumaira S., Vijayarathna S., Hemagirri M., Adnan M., Hassan I., Patel M., Gupta R., Shanmugapriya, Chen Y., Gopinath S.C. (2024). Plant bioactive compounds driven microRNAs (miRNAs): A potential source and novel strategy targeting gene and cancer therapeutics. Non-Coding RNA Res..

[79] Islam M.T., Jang N.H., Lee H.J. (2024). Natural Products as Regulators against Matrix Metalloproteinases for the Treatment of Cancer. Biomedicines.

[80] El Omari N., Bakrim S., Bakha M., Lorenzo J.M., Rebezov M., Shariati M.A., Aboulaghras S., Balahbib A., Khayrullin M., Bouyahya A. (2021). Natural Bioactive Compounds Targeting Epigenetic Pathways in Cancer: A Review on Alkaloids, Terpenoids, Quinones, and Isothiocyanates. Nutrients.

[81] Stylianou E. (2018). Epigenetics of chronic inflammatory diseases. J. Inflamm. Res..

[82] Hassan F.U., Rehman M.S., Khan M.S., Ali M.A., Javed A., Nawaz A., Yang C. (2019). Curcumin as an Alternative Epigenetic Modulator: Mechanism of Action and Potential Effects. Front. Genet..

[83] Seto E., Yoshida M. (2014). Erasers of histone acetylation: The histone deacetylase enzymes. Cold Spring Harb. Perspect. Biol..

[84] Rossetto D., Avvakumov N., Cote J. (2012). Histone phosphorylation: A chromatin modification involved in diverse nuclear events. Epigenetics.

[85] Pieniawska M., Iżykowska K. (2022). Role of Histone Deacetylases in T-Cell Development and Function. Int. J. Mol. Sci..

[86] Shu L., Khor T.O., Lee J.-H., Boyanapalli S.S.S., Huang Y., Wu T.-Y., Saw C.L.-L., Cheung K.-L., Kong A.-N.T. (2011). Epigenetic CpG Demethylation of the Promoter and Reactivation of the Expression of Neurog1 by Curcumin in Prostate LNCaP Cells. AAPS J..

[87] Koprinarova M., Schnekenburger M., Diederich M. (2016). Role of Histone Acetylation in Cell Cycle Regulation. Curr. Top. Med. Chem..

[88] Kleftogiannis D., Kalnis P., Arner E., Bajic V.B. (2017). Discriminative identification of transcriptional responses of promoters and enhancers after stimulus. Nucleic Acids Res..

[89] Ahmed A.U., Williams B.R., Hannigan G.E. (2015). Transcriptional Activation of Inflammatory Genes: Mechanistic Insight into Selectivity and Diversity. Biomolecules.

[90] Azzini E., Peña-Corona S.I., Hernández-Parra H., Chandran D., Saleena L.A.K., Sawikr Y., Peluso I., Dhumal S., Kumar M., Leyva-Gómez G. (2024). Neuroprotective and Anti-Inflammatory Effects of Curcumin in Alzheimer’s Disease: Targeting Neuroinflammation Strategies. Phytother. Res..

[91] Fabianowska-Majewska K., Kaufman-Szymczyk A., Szymanska-Kolba A., Jakubik J., Majewski G., Lubecka K. (2021). Curcumin from Turmeric Rhizome: A Potential Modulator of DNA Methylation Machinery in Breast Cancer Inhibition. Nutrients.

[92] Elmore S. (2007). Apoptosis: A review of programmed cell death. Toxicol. Pathol..

[93] Carrillo-Martinez E.J., Flores-Hernández F.Y., Salazar-Montes A.M., Nario-Chaidez H.F., Hernández-Ortega L.D. (2024). Quercetin, a Flavonoid with Great Pharmacological Capacity. Molecules.

[94] Primikyri A., Chatziathanasiadou M.V., Karali E., Kostaras E., Mantzaris M.D., Hatzimichael E., Shin J.S., Chi S.W., Briasoulis E., Kolettas E. (2014). Direct binding of Bcl-2 family proteins by quercetin triggers its pro-apoptotic activity. ACS Chem. Biol..

[95] Lu L., Hu W., Tian Z., Yuan D., Yi G., Zhou Y., Cheng Q., Zhu J., Li M. (2019). Developing natural products as potential anti-biofilm agents. Chin. Med..

[96] Preda V.G., Săndulescu O. (2019). Communication is the key: Biofilms, quorum sensing, formation and prevention. Discoveries.

[97] Roy R., Tiwari M., Donelli G., Tiwari V. (2018). Strategies for combating bacterial biofilms: A focus on anti-biofilm agents and their mechanisms of action. Virulence.

[98] Ciandrini E., Campana R., Federici S. (2014). In vitro activity of Carvacrol against titanium-adherent oral biofilms and planktonic cultures. Clin. Oral Investig..

[99] Wang Y., Liu B., Grenier D., Yi L. (2019). Regulatory Mechanisms of the LuxS/AI-2 System and Bacterial Resistance. Antimicrob. Agents Chemother..

[100] Deng Z., Hou K., Valencak T.G., Luo X.M., Liu J., Wang H. (2022). AI-2/LuxS Quorum Sensing System Promotes Biofilm Formation of Lactobacillus rhamnosus GG and Enhances the Resistance to Enterotoxigenic Escherichia coli in Germ-Free Zebrafish. Microbiol. Spectr..

[101] Poulin M.B., Kuperman L.L. (2021). Regulation of Biofilm Exopolysaccharide Production by Cyclic Di-Guanosine Monophosphate. Front. Microbiol..

[102] Burt S.A., Ojo-Fakunle V.T., Woertman J., Veldhuizen E.J. (2014). The natural antimicrobial carvacrol inhibits quorum sensing in *Chromobacterium violaceum* and reduces bacterial biofilm formation at sub-lethal concentrations. PLoS ONE.

[103] Shao H., Lamont R.J., Demuth D.R. (2007). Autoinducer 2 is required for biofilm growth of *Aggregatibacter* (*Actinobacillus*) *actinomycetemcomitans*. Infect. Immun..

[104] Cirano F.R., Casarin R.C.V., Ribeiro F.V., Casati M.Z., Pimentel S.P., Taiete T., Bernardi M.M. (2016). Effect of Resveratrol on periodontal pathogens during experimental periodontitis in rats. Braz. Oral Res..

[105] O’Connor D.J., Wong R.W., Rabie A.B. (2011). Resveratrol inhibits periodontal pathogens in vitro. Phytother. Res..

[106] Xie Q., Johnson B.R., Wenckus C.S., Fayad M.I., Wu C.D. (2012). Efficacy of Berberine, an Antimicrobial Plant Alkaloid, as an Endodontic Irrigant against a Mixed-culture Biofilm in an In Vitro Tooth Model. J. Endod..

[107] Zhao N., Isguven S., Evans R., Schaer T.P., Hickok N.J. (2023). Berberine disrupts staphylococcal proton motive force to cause potent anti-staphylococcal effects in vitro. Biofilm.

[108] Li C.-G., Yan L., Jing Y.-Y., Xu L.-H., Liang Y.-D., Wei H.-X., Hu B., Pan H., Zha Q.-B., Ouyang D.-Y. (2017). Berberine augments ATP-induced inflammasome activation in macrophages by enhancing AMPK signaling. Oncotarget.

[109] Marques C., Grenho L., Fernandes M.H., Costa Lima S.A. (2024). Improving the Antimicrobial Potency of Berberine for Endodontic Canal Irrigation Using Polymeric Nanoparticles. Pharmaceutics.

[110] Wu M., Brown A.C. (2021). Applications of Catechins in the Treatment of Bacterial Infections. Pathogens.

[111] Zhuang Y., Quan W., Wang X., Cheng Y., Jiao Y. (2024). Comprehensive Review of EGCG Modification: Esterification Methods and Their Impacts on Biological Activities. Foods.

[112] Sanders H.M., Kostelic M.M., Zak C.K., Marty M.T. (2022). Lipids and EGCG Affect α-Synuclein Association and Disruption of Nanodiscs. Biochemistry.

[113] Steinmann J., Buer J., Pietschmann T., Steinmann E. (2013). Anti-infective properties of epigallocatechin-3-gallate (EGCG), a component of green tea. Br. J. Pharmacol..

[114] Fournier-Larente J., Morin M.P., Grenier D. (2016). Green tea catechins potentiate the effect of antibiotics and modulate adherence and gene expression in *Porphyromonas gingivalis*. Arch. Oral Biol..

[115] Cai Y., Chen Z.B., Liu H., Xuan Y., Wang X.X., Luan Q.X. (2015). Green tea epigallocatechin-3-gallate alleviates *Porphyromonas gingivalis*-induced periodontitis in mice. Int. Immunopharmacol..

[116] Nie T., Zhang C., Huang A., Li P. (2018). Epigallocatechin Gallate-Mediated Cell Death Is Triggered by Accumulation of Reactive Oxygen Species Induced via the Cpx Two-Component System in Escherichia coli. Front. Microbiol..

[117] Muteeb G., Rehman M.T., Shahwan M., Aatif M. (2023). Origin of Antibiotics and Antibiotic Resistance, and Their Impacts on Drug Development: A Narrative Review. Pharmaceuticals.

[118] Lorca G., Ballestero D., Langa E., Pino-Otín M.R. (2024). Enhancing Antibiotic Efficacy with Natural Compounds: Synergistic Activity of Tannic Acid and Nerol with Commercial Antibiotics against Pathogenic Bacteria. Plants.

[119] Gaurav A., Bakht P., Saini M., Pandey S., Pathania R. (2023). Role of bacterial efflux pumps in antibiotic resistance, virulence, and strategies to discover novel efflux pump inhibitors. Microbiology.

[120] Vaou N., Stavropoulou E., Voidarou C., Tsakris Z., Rozos G., Tsigalou C., Bezirtzoglou E. (2022). Interactions between Medical Plant-Derived Bioactive Compounds: Focus on Antimicrobial Combination Effects. Antibiotics.

[121] Varghese M.K., Nagarathna D.V., Litty Scariya L.S. (2014). Curcumin and metronidazole in periodontal therapy. Int. J. Res. Ayurveda Pharm..

[122] Ravishankar P., Kumar Y.P., Anila E., Chakraborty P., Malakar M., Maha-lakshmi R. (2017). Effect of local application of curcumin and ornidazole gel in chronic periodontitis patients. Int. J. Pharm. Investig..

[123] Qin T., Chen K., Xi B., Pan L., Xie J., Lu L., Liu K. (2023). In Vitro Antibiofilm Activity of Resveratrol against *Aeromonas hydrophila*. Antibiotics.

[124] Mirghani R., Saba T., Khaliq H., Mitchell J., Do L., Chambi L., Diaz K., Kennedy T., Alkassab K., Huynh T. (2022). Biofilms: Formation, drug resistance and alternatives to conventional approaches. AIMS Microbiol..

[125] Peng Q., Tang X., Dong W., Zhi Z., Zhong T., Lin S., Ye J., Qian X., Chen F., Yuan W. (2023). Carvacrol inhibits bacterial polysaccharide intracellular adhesin synthesis and biofilm formation of mucoid Staphylococcus aureus: An in vitro and in vivo study. RSC Adv..

[126] Soumya E.A., Saad I.K., Hassan L., Ghizlane Z., Hind M., Adnane R. (2011). Carvacrol and thymol components inhibiting Pseudomonas aeruginosa adherence and biofilm formation. Afr. J. Microbiol. Res..

[127] Wijesundara N.M., Lee S.F., Cheng Z., Davidson R., Langelaan D.N., Rupasinghe H.P.V. (2022). Bactericidal Activity of Carvacrol against Streptococcus pyogenes Involves Alteration of Membrane Fluidity and Integrity through Interaction with Membrane Phospholipids. Pharmaceutics.

[128] Limoli D.H., Jones C.J., Wozniak D.J. (2015). Bacterial Extracellular Polysaccharides in Biofilm Formation and Function. Microbiol. Spectr..

[129] Campoccia D., Montanaro L., Arciola C.R. (2021). Extracellular DNA (eDNA). A Major Ubiquitous Element of the Bacterial Biofilm Architecture. Int. J. Mol. Sci..

[130] Serrage H.J., Jepson M.A., Rostami N., Jakubovics N.S., Nobbs A.H. (2021). Understanding the Matrix: The Role of Extracellular DNA in Oral Biofilms. Front. Oral Health.

[131] Fu J., Zhang Y., Lin S., Zhang W., Shu G., Lin J., Li H., Xu F., Tang H., Peng G. (2021). Strategies for Interfering with Bacterial Early Stage Biofilms. Front. Microbiol..

[132] Zhang Q., Ma Q., Wang Y., Wu H., Zou J. (2021). Molecular mechanisms of inhibiting glucosyltransferases for biofilm formation in *Streptococcus mutans*. Int. J. Oral Sci..

[133] Hu Z., Guan Y., Hu W., Xu Z., Ishfaq M. (2022). An overview of pharmacological activities of baicalin and its aglycone baicalein: New insights into molecular mechanisms and signaling pathways. Iran. J. Basic Med. Sci..

[134] Balducci E., Papi F., Capialbi D.E., Del Bino L. (2023). Polysaccharides’ Structures and Functions in Biofilm Architecture of Antimicrobial-Resistant (AMR) Pathogens. Int. J. Mol. Sci..

[135] Ma C., Mei C., Liu J., Li H., Jiao M., Hu H., Zhang Y., Xiong J., He Y., Wei W. (2024). Effect of baicalin on eradicating biofilms of bovine milk derived *Acinetobacter lwoffii*. BMC Vet. Res..

[136] Luo J., Dong B., Wang K., Cai S., Liu T., Cheng X., Lei D., Chen Y., Li Y., Kong J. (2017). Baicalin inhibits biofilm formation, attenuates the quorum sensing-controlled virulence and enhances *Pseudomonas aeruginosa* clearance in a mouse peritoneal implant infection model. PLoS ONE.

[137] Asma S.T., Imre K., Morar A., Herman V., Acaroz U., Mukhtar H., Arslan-Acaroz D., Shah S.R.A., Gerlach R. (2022). An Overview of Biofilm Formation-Combating Strategies and Mechanisms of Action of Antibiofilm Agents. Life.

[138] Yang B., Pang X., Li Z., Chen Z., Wang Y. (2021). Immunomodulation in the Treatment of Periodontitis: Progress and Perspectives. Front. Immunol..

[139] Feng Y., Chen Z., Tu S.Q., Wei J.M., Hou Y.L., Kuang Z.L., Kang X.N., Ai H. (2022). Role of Interleukin-17A in the Pathomechanisms of Periodontitis and Related Systemic Chronic Inflammatory Diseases. Front. Immunol..

[140] Ghoushi E., Poudineh M., Parsamanesh N., Jamialahmadi T., Sahebkar A. (2024). Curcumin as a regulator of Th17 cells: Unveiling the mechanisms. Food Chem..

[141] Sivani B.M., Azzeh M., Patnaik R., Pantea Stoian A., Rizzo M., Banerjee Y. (2022). Reconnoitering the Therapeutic Role of Curcumin in Disease Prevention and Treatment: Lessons Learnt and Future Directions. Metabolites.

[142] Shakoor H., Feehan J., Apostolopoulos V., Platat C., Al Dhaheri A.S., Ali H.I., Ismail L.C., Bosevski M., Stojanovska L. (2021). Immunomodulatory Effects of Dietary Polyphenols. Nutrients.

[143] Cui H., Wang N., Li H., Bian Y., Wen W., Kong X., Wang F. (2024). The dynamic shifts of IL-10-producing Th17 and IL-17-producing Treg in health and disease: A crosstalk between ancient “Yin-Yang” theory and modern immunology. Cell Commun. Signal.

[144] Jiang H., Ni J., Hu L., Xiang Z., Zeng J., Shi J., Chen Q., Li W. (2023). Resveratrol May Reduce the Degree of Periodontitis by Regulating ERK Pathway in Gingival-Derived MSCs. Int. J. Mol. Sci..

[145] Ren M., Zhao Y., He Z., Lin J., Xu C., Liu F., Hu R., Deng H., Wang Y. (2021). Baicalein inhibits inflammatory response and promotes osteogenic activity in periodontal ligament cells challenged with lipopolysaccharides. BMC Complement. Med. Ther..

[146] Yang J., Yang X., Li M. (2012). Baicalin, a natural compound, promotes regulatory T cell differentiation. BMC Complement. Altern. Med..

[147] Liu C., Li Y., Chen Y., Huang S., Wang X., Luo S., Su Y., Zhou L., Luo X. (2020). Baicalein Restores the Balance of Th17/Treg Cells via Aryl Hydrocarbon Receptor to Attenuate Colitis. Mediat. Inflamm..

[148] González-Rodríguez M., Edjoudi D.A., Cordero-Barreal A., Farrag M., Varela-García M., Torrijos-Pulpón C., Ruiz-Fernández C., Capuozzo M., Ottaiano A., Lago F. (2023). Oleocanthal, an Antioxidant Phenolic Compound in Extra Virgin Olive Oil (EVOO): A Comprehensive Systematic Review of Its Potential in Inflammation and Cancer. Antioxidants.

[149] Rodríguez-Agurto A., Bravo M., Magán-Fernandez A., López-Toruño A., Muñoz R., Ferrer J., Mesa F. (2023). Randomized clinical trial on the clinical effects of a toothpaste containing extra virgin olive oil, xylitol, and betaine in gingivitis. Sci. Rep..

[150] Mehrotra P., Ravichandran K.S. (2022). Drugging the efferocytosis process: Concepts and opportunities. Nat. Rev. Drug Discov..

[151] Miralda I., Uriarte S.M. (2021). Periodontal Pathogens’ strategies disarm neutrophils to promote dysregulated inflammation. Mol. Oral Microbiol..

[152] Li B., Xin Z., Gao S., Li Y., Guo S., Fu Y., Xu R., Wang D., Cheng J., Liu L. (2023). SIRT6-regulated macrophage efferocytosis epigenetically controls inflammation resolution of diabetic periodontitis. Theranostics.

[153] Ge Y., Huang M., Yao Y.M. (2022). Efferocytosis and Its Role in Inflammatory Disorders. Front. Cell Dev. Biol..

[154] Loh W., Vermeren S. (2022). Anti-Inflammatory Neutrophil Functions in the Resolution of Inflammation and Tissue Repair. Cells.

[155] Guan X., Wang Y., Li W., Mu W., Tang Y., Wang M., Seyam A., Yang Y., Pan L., Hou T. (2024). The Role of Macrophage Efferocytosis in the Pathogenesis of Apical Periodontitis. Int. J. Mol. Sci..

[156] Wang J., Hashimoto Y., Hiemori-Kondo M., Nakamoto A., Sakai T., Ye W., Abe-Kanoh N. (2024). Resveratrol and piceid enhance efferocytosis by increasing the secretion of MFG-E8 in human THP-1 macrophages. Biosci. Biotechnol. Biochem..

[157] Parinot C., Chatagnon J., Rieu Q., Roux S., Néel D., Hamieh F., Nandrot E.F. (2024). Gas6 and Protein S Ligands Cooperate to Regulate MerTK Rhythmic Activity Required for Circadian Retinal Phagocytosis. Int. J. Mol. Sci..

[158] Deng J., Golub L.M., Lee H.-M., Bhatt H.-D., Johnson F., Xu T.-M., Gu Y. (2023). A novel modified-curcumin 2.24 resolves inflammation by promoting M2 macrophage polarization. Sci. Rep..

[159] Al-Kattan R. (2024). The role of curcumin in periodontal therapy: An update. Funct. Foods Health Dis..

[160] Silverstein R.L., Febbraio M. (2009). CD36, a scavenger receptor involved in immunity, metabolism, angiogenesis, and behavior. Sci. Signal..

[161] Bae H.B., Zmijewski J.W., Deshane J.S., Tadie J.M., Chaplin D.D., Takashima S., Abraham E. (2011). AMP-activated protein kinase enhances the phagocytic ability of macrophages and neutrophils. FASEB J..

[162] Felix F.B., Dias J., Vago J.P., Martins D.G., Beltrami V.A., Fernandes D.d.O., dos Santos A.C.P.M., Queiroz-Junior C.M., de Sousa L.P., Amaral F.A. (2023). Blocking the HGF-MET pathway induces resolution of neutrophilic inflammation by promoting neutrophil apoptosis and efferocytosis. Pharmacol. Res..

[163] Ramesh P., Jagadeesan R., Sekaran S., Dhanasekaran A., Vimalraj S. (2021). Flavonoids: Classification, Function, and Molecular Mechanisms Involved in Bone Remodelling. Front. Endocrinol..

[164] Inchingolo A.D., Inchingolo A.M., Malcangi G., Avantario P., Azzollini D., Buongiorno S., Viapiano F., Campanelli M., Ciocia A.M., De Leonardis N. (2022). Effects of Resveratrol, Curcumin and Quercetin Supplementation on Bone Metabolism—A Systematic Review. Nutrients.

[165] Cao L., Wang J., Zhang Y., Tian F., Wang C. (2022). Osteoprotective effects of flavonoids: Evidence from in vivo and in vitro studies (Review). Mol. Med. Rep..

[166] Kugaji M.S., Kumbar V.M., Peram M.R., Patil S., Bhat K.G., Diwan P.V. (2019). Effect of Resveratrol on Biofilm Formation and Virulence Factor Gene Expression of *Porphyromonas gingivalis* in Periodontal Disease. APMIS.

[167] Kumbar V.M., Peram M.R., Kugaji M.S., Shah T., Patil S.P., Muddapur U.M., Bhat K.G. (2021). Effect of Curcumin on Growth, Biofilm Formation and Virulence Factor Gene Expression of *Porphyromonas gingivalis*. Odontology.

[168] Guimarães M.R., Coimbra L.S., De Aquino S.G., Spolidorio L.C., Kirkwood K.L., Rossa C. (2011). Potent Anti-inflammatory Effects of Systemically Administered Curcumin Modulate Periodontal Disease In Vivo. J. Periodontal Res..

[169] Abdel-Fatah R., Mowafey B., Baiomy A., Elmeadawy S. (2023). Efficacy of curcumin gel as an adjunct to scaling and root planing on salivary procalcitonin level in the treatment of patients with chronic periodontitis: A randomized controlled clinical trial. BMC Oral Health.

[170] Anitha V., Rajesh P., Shanmugam M., Priya B., Prabhu S., Shivakumar V. (2015). Comparative evaluation of natural curcumin and synthetic chlorhexidine in the management of chronic periodontitis as a local drug delivery: A clinical and microbiological study. Indian J. Dent. Res..

[171] Peng Y., Ao M., Dong B., Jiang Y., Yu L., Chen Z., Hu C., Xu R. (2021). Anti-inflammatory effects of curcumin in inflammatory diseases: Status, limitations, and countermeasures. Drug Des. Dev. Ther..

[172] Zhou Y., Guan X., Zhu W., Liu Z., Wang X., Yu H., Wang H. (2013). Capsaicin inhibits *Porphyromonas gingivalis* growth, biofilm formation, gin-givomucosal inflammatory cytokine secretion, and in vitro osteoclasto-genesis. Eur. J. Clin. Microbiol. Infect. Dis..

[173] Wattel A., Kamel S., Prouillet C., Petit J.P., Lorget F., Offord E., Brazier M. (2004). Flavonoid quercetin decreases osteoclastic differentiation induced by RANKL via a mechanism involving NF-κB and AP-1. J. Cell. Biochem..

[174] Yang S.-Y., Hu Y., Zhao R., Zhou Y.-N., Zhuang Y., Zhu Y., Ge X.-L., Lu T.-W., Lin K.-L., Xu Y.-J. (2024). Quercetin-loaded mesoporous nano-delivery system remodels osteoimmune microenvironment to regenerate alveolar bone in periodontitis via the miR-21a-5p/PDCD4/NF-κB pathway. J. Nanobiotechnol..

[175] Anuradha B., Bai Y., Sailaja S., Sudhakar J., Priyanka M., Deepika V. (2015). Evaluation of anti-inflammatory effects of curcumin gel as an adjunct to scaling and root planing: A clinical study. J. Int. Oral Health.

[176] Singh P., Gupta R., Siddharth M., Sinha A., Shree S., Sharma K. (2020). A comparative evaluation of subgingivally delivered 2% curcumin and 0.2% chlorhexidine gel adjunctive to scaling and root planing in chronic periodontitis. J. Contemp. Dent. Pract..

[177] Botelho M.A., dos Santos R.A., Martins J.G., Carvalho C.O., Paz M.C., Azenha C., Ruela R.S., Queiroz D.B., Ruela W.S., Marinho G. (2009). Comparative effect of an essential oil mouthrinse on plaque, gingivitis and salivary Streptococcus mutans levels: A double blind randomized study. Phytother. Res..

[178] Taleghani F., Rezvani G., Birjandi M., Valizadeh M. (2018). Impact of green tea intake on clinical improvement in chronic periodontitis: A randomized clinical trial. J. Stomatol. Oral Maxillofac. Surg..

[179] Sarin S., Marya C., Nagpal R., Oberoi S.S., Rekhi A. (2015). Preliminary clinical evidence of the antiplaque, antigingivitis efficacy of a mouthwash containing 2% green tea—A randomised clinical trial. Oral Health Prev. Dent..

[180] Moeintaghavi A., Shabzendedar M., Parissay I., Makarem A., Orafaei H., Hosseinnezhad M. (2012). Effect of Berberine Gel on Periodontal Inflammation: Clinical and Histological. J. Periodontol. Implant. Dent..

[181] Elburki M.S., Moore D.D., Terezakis N.G., Zhang Y., Lee H.M., Johnson F., Golub L.M. (2017). A novel chemically modified curcumin reduces inflammation-mediated connective tissue breakdown in a rat model of diabetes: Periodontal and systemic effects. J. Periodontal Res..

[182] Gao S., Hu M. (2010). Bioavailability challenges associated with development of anti-cancer phenolics. Mini Rev. Med. Chem..

[183] Bertoncini-Silva C., Vlad A., Ricciarelli R., Giacomo Fassini P., Suen V.M.M., Zingg J.-M. (2024). Enhancing the Bioavailability and Bioactivity of Curcumin for Disease Prevention and Treatment. Antioxidants.

[184] Mehmood S., Maqsood M., Mahtab N., Khan M.I., Sahar A., Zaib S., Gul S. (2022). Epigallocatechin gallate: Phytochemistry, bioavailability, utilization challenges, and strategies. J. Food Biochem..

[185] Lee S.J., Krauthauser C., Maduskuie V., Fawcett P.T., Olson J.M., Rajasekaran S.A. (2011). Curcumin-induced HDAC inhibition and attenuation of medulloblastoma growth in vitro and in vivo. BMC Cancer.

[186] Li J., Chai R., Chen Y., Zhao S., Bian Y., Wang X. (2022). Curcumin Targeting Non-Coding RNAs in Colorectal Cancer: Therapeutic and Biomarker Implications. Biomolecules.

[187] Gilyazova I., Asadullina D., Kagirova E., Sikka R., Mustafin A., Ivanova E., Bakhtiyarova K., Gilyazova G., Gupta S., Khusnutdinova E. (2023). MiRNA-146a—A Key Player in Immunity and Diseases. Int. J. Mol. Sci..

[188] Shaito A., Al-Mansoob M., Ahmad S.M.S., Haider M.Z., Eid A.H., Posadino A.M., Pintus G., Giordo R. (2023). Resveratrol-Mediated Regulation of Mitochondria Biogenesis-associated Pathways in Neurodegenerative Diseases: Molecular Insights and Potential Therapeutic Applications. Curr. Neuropharmacol..

[189] Dembic M., Andersen H.S., Bastin J., Doktor T.K., Corydon T.J., Sass J.O., Costa A.L., Djouadi F., Andresen B.S. (2019). Next generation sequencing of RNA reveals novel targets of resveratrol with possible implications for Canavan disease. Mol. Genet. Metab..

[190] Nishigaki A., Kido T., Kida N., Kakita-Kobayashi M., Tsubokura H., Hisamatsu Y., Okada H. (2020). Resveratrol protects mitochondrial quantity by activating SIRT1/PGC-1α expression during ovarian hypoxia. Reprod. Med. Biol..

[191] Zhang X., Jin Y., Wang Q., Jian F., Li M., Long H., Lai W. (2020). Autophagy-mediated regulation patterns contribute to the alterations of the immune microenvironment in periodontitis. Aging.

[192] Bullon P., Cordero M.D., Quiles J.L., del Carmen Ramirez-Tortosa M., Gonzalez-Alonso A., Alfonsi S., García-Marín R., de Miguel M., Battino M. (2012). Autophagy in periodontitis patients and gingival fibroblasts: Unraveling the link between chronic diseases and inflammation. BMC Med..

[193] Jin F., Li J., Zhao C., Gu L., Pu M., Jiang S., Liang M., Zhao Y., Shen J., Agabuwei A. (2024). Quercetin alleviates kidney damage caused by mercury Chloride: The protective effects of quercetin on autophagy and inflammation were studied based on TRIM32/TLR4/LC3 pathway. Toxicon.

[194] Ge J., Li M., Yao J., Guo J., Li X., Li G., Han X., Li Z., Liu M., Zhao J. (2024). The potential of EGCG in modulating the oral-gut axis microbiota for treating inflammatory bowel disease. Phytomedicine.

[195] Pérez-Burillo S., Navajas-Porras B., López-Maldonado A., Hinojosa-Nogueira D., Pastoriza S., Rufián-Henares J.Á. (2021). Green Tea and Its Relation to Human Gut Microbiome. Molecules.

[196] Hashim N.T., Babiker R., Rahman M.M., Chaitanya N.C.S.K., Mohammed R., Dasnadi S.P., Gismalla B.G. (2024). Gum Arabic as a potential candidate in quorum quenching and treatment of periodontal diseases. Front. Oral Health.

[197] Wen Y., Wang Y., Zhao C., Zhao B., Wang J. (2023). The Pharmacological Efficacy of Baicalin in Inflammatory Diseases. Int. J. Mol. Sci..

[198] An H.J., Lee J.Y., Park W. (2022). Baicalin Modulates Inflammatory Response of Macrophages Activated by LPS via Calcium-CHOP Pathway. Cells.

[199] Cai X., Shi Y., Dai Y., Wang F., Chen X., Li X. (2022). Baicalin clears inflammation by enhancing macrophage efferocytosis via inhibition of RhoA/ROCK signaling pathway and regulating macrophage polarization. Int. Immunopharmacol..

[200] Lu L., Wang J., Qin T., Chen K., Xie J., Xi B. (2023). Carvacrol Inhibits Quorum Sensing in Opportunistic Bacterium Aeromonas hydrophila. Microorganisms.

[201] Wright P.P., Ramachandra S.S. (2022). Quorum Sensing and Quorum Quenching with a Focus on Cariogenic and Periodontopathic Oral Biofilms. Microorganisms.

[202] Jha N.K., Sharma C., Hashiesh H.M., Arunachalam S., Meeran M.N., Javed H., Patil C.R., Goyal S.N., Ojha S. (2021). β-Caryophyllene, A Natural Dietary CB2 Receptor Selective Cannabinoid can be a Candidate to Target the Trinity of Infection, Immunity, and Inflammation in COVID-19. Front. Pharmacol..

[203] Turcotte C., Blanchet M.R., Laviolette M., Flamand N. (2016). The CB2 receptor and its role as a regulator of inflammation. Cell. Mol. Life Sci..

[204] Bunggulawa E.J., Wang W., Yin T., Wang N., Durkan C., Wang Y., Wang G. (2018). Recent advancements in the use of exosomes as drug delivery systems. J. Nanobiotechnol.

[205] Yoo H.J., Jwa S.K. (2019). Efficacy of β-caryophyllene for periodontal disease related factors. Arch. Oral Biol..

[206] Yang S.C., Alalaiwe A., Lin Z.C., Lin Y.C., Aljuffali I.A., Fang J.Y. (2022). Anti-Inflammatory microRNAs for Treating Inflammatory Skin Diseases. Biomolecules.

[207] Grieco G.E., Brusco N., Licata G., Nigi L., Formichi C., Dotta F., Sebastiani G. (2019). Targeting microRNAs as a Therapeutic Strategy to Reduce Oxidative Stress in Diabetes. Int. J. Mol. Sci..

[208] Song J., Wu Q., Jiang J., Sun D., Wang F., Xin B., Cui Q. (2020). Berberine reduces inflammation of human dental pulp fibroblast via miR-21/KBTBD7 axis. Arch. Oral Biol..

[209] Gao Z.-S., Zhang C.-J., Xia N., Tian H., Li D.-Y., Lin J.-Q., Mei X.-F., Wu C. (2021). Berberine-loaded M2 macrophage-derived exosomes for spinal cord injury therapy. Acta Biomater..

[210] Nair A., Mallya R., Suvarna V., Khan T.A., Momin M., Omri A. (2022). Nanoparticles-Attractive Carriers of Antimicrobial Essential Oils. Antibiotics.

[211] Movahedi F., Nirmal N., Wang P., Jin H., Grøndahl L., Li L. (2024). Recent advances in essential oils and their nanoformulations for poultry feed. J. Anim. Sci. Biotechnol..

[212] Sharifi-Rad J., El Rayess Y., Rizk A.A., Sadaka C., Zgheib R., Zam W., Sestito S., Rapposelli S., Neffe-Skocińska K., Zielińska D. (2011). Turmeric and Its Major Compound Curcumin on Health: Bioactive Effects and Safety Profiles for Food, Pharmaceutical, Biotechnological and Medicinal Applications. Front. Pharmacol..

[213] Sakano K., Kawanishi S. (2022). Metal-mediated DNA damage induced by curcumin in the presence of human cytochrome P450 isozymes. Arch. Biochem. Biophys..

[214] National Toxicology Program (1993). NTP Toxicology and Carcinogenesis Studies of Turmeric Oleoresin (CAS No. 8024-37-1) (Major Component 79–85% Curcumin, CAS No. 458-37-7) in F344/N Rats and B6C3F1 Mice (Feed Studies). Natl. Toxicol. Program Tech. Rep. Ser..

[215] Miao Y., Sun X., Gao G., Jia X., Wu H., Chen Y., Huang L. (2019). Evaluation of (−)-epigallocatechin-3-gallate (EGCG)-induced cytotoxicity on astrocytes: A potential mechanism of calcium overloading-induced mitochondrial dysfunction. Toxicol. Vitr..

[216] Olivares-Marin I.K., González-Hernández J.C., Madrigal-Perez L.A. (2019). Resveratrol cytotoxicity is energy-dependent. J. Food Biochem..

[217] Cho S.J., Jung U.J., Choi M.S. (2012). Differential effects of low-dose resveratrol on adiposity and hepatic steatosis in diet-induced obese mice. Br. J. Nutr..

[218] Lambert J.D., Kennett M.J., Sang S., Reuhl K.R., Ju J., Yang C.S. (2010). Hepatotoxicity of high oral dose (−)-epigallocatechin-3-gallate in mice. Food Chem. Toxicol..

[219] Fan Y.C., Chen C.N., Lin C.Y., Tsai E.M., Chan W.H. (2014). Epigallocatechin gallate induces embryonic toxicity in mouse blastocysts through apoptosis. Drug Chem. Toxicol..

